# The effect of carbohydrate intake on glycaemic control in individuals with type 1 diabetes: a randomised, open-label, crossover trial

**DOI:** 10.1016/j.lanepe.2023.100799

**Published:** 2023-12-19

**Authors:** Sofia Sterner Isaksson, Arndís F. Ólafsdóttir, Simon Ivarsson, Henrik Imberg, Eva Toft, Sara Hallström, Ulf Rosenqvist, Marie Ekström, Marcus Lind

**Affiliations:** a Department of Molecular and Clinical Medicine, Sahlgrenska Academy, University of Gothenburg, Gothenburg, Sweden; b Department of Medicine, NU Hospital Group, Uddevalla, Sweden; c Department of Medicine, Sahlgrenska University Hospital, Gothenburg, Sweden; d Statistiska Konsultgruppen, Gothenburg, Sweden; e Department of Mathematical Sciences, Chalmers University of Technology and University of Gothenburg, Gothenburg, Sweden; f Department of Medicine, Ersta Hospital, Stockholm, Sweden; g Department of Clinical Education and Science, Södersjukhuset, Karolinska Institute, Stockholm, Sweden; h Department of Internal Medicine, Motala Hospital, Motala, Sweden

**Keywords:** Type 1 diabetes mellitus, Glycaemic control, Continuous glucose monitoring, Medical nutrition therapy, Carbohydrates

## Abstract

**Background:**

Few studies have examined the effects of lower carbohydrate diets on glucose control in persons with type 1 diabetes (T1D). The objective of the study was to investigate whether a moderate carbohydrate diet improves glucose control in persons with T1D.

**Methods:**

A randomised, multicentre, open-label, crossover trial over 12 weeks. There were 69 individuals assessed for eligibility, 54 adults with T1D and HbA1c ≥ 58 mmol/mol (7.5%) were randomised. Interventions were moderate carbohydrate diet versus traditional diet (30 vs 50% of total energy from carbohydrates) over four weeks, with a four-week wash-out period between treatments. Masked continuous glucose monitoring was used to evaluate effects on glucose control. The primary endpoint was the difference in mean glucose levels between the last 14 days of each diet phase.

**Findings:**

50 individuals were included in the full analysis set with a mean baseline HbA1c of 69 mmol/mol (8.4%), BMI 29 kg/m^2^, age of 48 years, and 50% were female. The difference in mean glucose levels between moderate carbohydrate and traditional diet was −0.6 mmol/L, 95% CI −0.9 to −0.3, p < 0.001. Time in range increased during moderate carbohydrate diet by 4.7% (68 min/24 h) (95% CI 1.3 to 8.0), p = 0.008. Time above range (>10 mmol/L) decreased by 5.9% (85 min/24 h), 95% CI −9.6 to −2.2, p = 0.003. There were no significant differences in the standard deviation of glucose levels (95% CI −0.3 to 0.0 mmol/L, p = 0.15) or hypoglycaemia in the range <3.9 mmol/L (95% CI −0.4 to 2.9%, p = 0.13) and <3.0 mmol/L (95% CI −0.4 to 1.6%, p = 0.26). Four participants withdrew, none because of adverse events. There were no serious adverse events including severe hypoglycaemia and ketoacidosis. Mean ketone levels were 0.17 (SD 0.14) mmol/L during traditional and 0.18 (SD 0.13) mmol/L during moderate carbohydrate diet (p = 0.02).

**Interpretation:**

A moderate carbohydrate diet is associated with decreases in mean glucose levels and time above range and increases in time in range without increased risk of hypoglycaemia or ketoacidosis compared with a traditional diet in individuals with T1D.

**Funding:**

The Healthcare Board, 10.13039/100007212Region Västra Götaland, The Dr P Håkansson Foundation and the Swedish state under the agreement between the Swedish government and the county councils, the ALF-agreement [ALFGBG-966173].

**Research in context**

**Evidence before this study**

We searched PubMed with end date on May 15, 2017, using the search terms “carbohydrate restriction”, “low carbohydrate diet”, “LCD” and “type 1 diabetes” in combinations. The titles and/or abstracts were screened and selected manually. Very few studies were found although the ones found were indicating positive effects on glucose control. Concerns about elevated risk of dyslipidaemia, hypoglycaemia and diabetic ketoacidosis also existed. The studies found were either observational, small, not randomised, or lacked control groups. An update search in PubMed on May 15, 2023, using the same search terms did not reveal any new relevant evidence regarding glucose control or safety from larger randomised studies.

**Added value of this study**

In this randomised, crossover trial over 12 weeks including 54 adults with type 1 diabetes the primary outcome mean glucose level was significantly reduced by −0.6 mmol/L (−11 mg/dL) with moderate restricted carbohydrate diet compared to traditional diet. Other endpoints showed more time in range (3.9–10.0 mmol/L), time in tighter ranges (3.9–7.8 and 3.5–7.8 mmol/L), and less time with high and very high glucose levels as well as increased treatment satisfaction. There was no increase in time in hypoglycaemia or cardiovascular risk factors such as lipids and blood pressure, no ketoacidosis, severe elevated ketone levels, or serious adverse events during the trial.

**Implications of all the available evidence**

This study shows that a moderate carbohydrate diet is more effective than a traditional diet with a higher amount of carbohydrates in terms of decreasing mean glucose levels and time above range and increasing time in range and treatment satisfaction without increased risk of hypoglycaemia, dyslipidaemia, or ketoacidosis in individuals with type 1 diabetes. These results show that a healthy moderate carbohydrate diet can be considered as a safe and effective treatment option for individuals with type 1 diabetes which extends possibilities for more differentiated diabetes care and provide further options to individualising diet treatment.

## Introduction

Diet is important to reaching glycaemic targets for persons with type 1 diabetes (T1D).^1^, ^2^, ^3^ Carbohydrate counting is used to calculate insulin dose/bolus sizes based on insulin-to-carbohydrate ratio and other parameters, although data on this methods effectiveness on glucose control are conflicting.^4^, ^5^, ^6^ Various types of diets and specific food groups have demonstrated protective effects on cardiovascular risk and are incorporated into dietary guidelines for individuals with diabetes. However, these recommendations are primarily derived from studies focused on type 2 diabetes.^1^, ^2^, ^3^

Reaching glycaemic targets is associated with lower risk of complications and mortality in persons with T1D,^7^^,^^8^ yet mortality remains considerably higher despite novel diabetes therapies.^8^, ^9^, ^10^

Predicting the exact amount of insulin to offset carbohydrates is difficult because many factors affect this relationship such as intraindividual variation in the uptake of insulin^11^^,^^12^ and variations in physical activity.^13^ Reducing carbohydrate intake has the theoretical potential to reduce glucose levels in certain individuals with T1D by mitigating glycaemic peaks, even if the insulin dosage is not entirely calibrated.

Some individuals with T1D experiment with low carbohydrate diets even though evidence regarding safety and efficacy is lacking and healthcare has discouraged them out of concern for diabetic ketoacidosis. Few studies have been conducted and results are conflicting.^14^ Observational studies have shown positive associations between lower carbohydrate intake and HbA1c.^15^, ^16^, ^17^ Studies showing positive effects on glycaemic control included few participants,^18^, ^19^, ^20^, ^21^ were not randomised, or lacked control groups.^21^^,^^22^ Negative effects including dyslipidaemia and greater hypoglycaemic episodes have also been shown.^23^ Despite several studies in type 2 diabetes showing a moderate carbohydrate diet as safe and effective to reaching treatment goals,^1^ results in T1D are unknown.

The aim of this study was to determine the effects of a moderate versus traditional diet on glycaemic control and risk of ketoacidosis in individuals with T1D.

## Methods

### Design

Randomised, open-label, crossover trial at four diabetes specialty clinics in Sweden. Study design and methods have previously been described in detail.^24^ The study protocol and CONSORT checklist is in eMethods1 and eMethods2, respectively. The study was approved by the regional ethics committee of Gothenburg, Sweden (No. 473-17). All participants provided written informed consent. The study was registered at clinicaltrials.gov, ID: NCT03400618. https://www.clinicaltrials.gov/ct2/show/NCT03400618.

### Trial procedures and evaluation period

Adults with T1D and HbA1c levels ≥58 mmol/mol (7.5%) were included in the trial. Other inclusion and exclusion criteria are given in eTable 1. After a run-in period of 2–4 weeks, those meeting inclusion criteria performed masked continuous glucose monitoring (CGM) for 2 weeks and completed 4 days of food recording, followed by 1:1 randomisation to moderate carbohydrate or traditional diet for 4 weeks with a 4-week wash-out period in between. Participants were randomised on site using a centralised web system with random permuted blocks of varying sizes. This was an open-label trial. It was not possible to use a blinded design and both participants and caregivers were aware of which diet participants were assigned to. All participants were given a coded subject ID and had 6 visits to diabetes clinics. Telephone follow-up was performed during diet interventions at days 1, 4, 7, and 14. During the first 2 weeks diabetes nurses and dietitians assisted with insulin and diet adjustments, respectively.^24^ Evaluations were completed during the last 2 weeks of each treatment phase when participants did not receive any clinical support.

### Dietary interventions

Moderate carbohydrate diet included approximately 30 percent of total energy from carbohydrates, 20 from proteins, and 50 from fat, and included a lot of vegetables, unsaturated oils, nuts, and carbohydrates were mainly from wholegrains and low glycaemic index foods. Traditional diet included approximately 50 percent of total energy from carbohydrates, 20 from proteins, and 30 from fat, and included foods according to guidelines.^1^, ^2^, ^3^ Both diets have been described in detail^24^ and were individualised by a dietitian to fit in terms of energy and macronutrient intake and in line with current nutrition guidelines. All participants were encouraged to consume carbohydrates evenly throughout the day, and diets were calculated to maintain energy balance and avoid weight changes. Participants were encouraged to maintain diet in accordance with protocol. All diet materials were developed by a dietitian and included recipes, meal examples with carbohydrate content, complete day menus, and ideas for foods, snacks, and meals. The same material was used at all study sites but was individualised by the local dietitian depending on individual energy demands and personal preferences. An example of a daily diet plan with the moderate carbohydrate diet is provided online (eTable 2A–C).

All participants recorded food intake in a 4-day food diary on 3 separate occasions beginning with the run-in period to provide data for the dietitian to make calculations and understand food preferences to create individual diet plans and during the last two weeks of each intervention period to measure adherence to diets. Protocol compliance and adherence, blood glucose, and insulin dosage was checked at each contact. Participants were encouraged to maintain regular physical activity level throughout the study which was measured by questionnaire.

### Continuous glucose monitoring

All participants used a masked CGM system (Freestyle Libre Pro, Abbott Diabetes Care) starting with run-in and then continuously during the 14–16-week study period. Participants using CGM or intermittent scanned CGM in usual diabetes care continued use. CGM system data were collected at all clinical visits, and data from the last 14 calendar days in each diet period were used in efficacy analyses. Masked CGM data were primarily used if available in both treatment phases. If data were lacking or sporadic it was prespecified that data from participants’ own CGM device would be used, which was the case in 6 participants (for definitions see the statistical analysis plan provided in eMethods3). The CGM devices were: 2 Freestyle Libre, and 1 Freestyle Libre 2 (Abbott Diabetes Care), 1 Dexcom G5, and 2 Dexcom G6 (Dexcom Inc.). All deviations were documented prior to database lock.

### Other measurements

HbA1c was measured at baseline. Blood lipids (total, LDL and HDL cholesterol, apolipoproteins, triglycerides), blood pressure, and weight were measured before and after each treatment period. Blood ketones were measured and recorded by participants in a diary twice a week at morning and evening during the two diet periods using a ketone measurement device. Hypoglycaemia confidence scale (HCS)^25^ and diabetes treatment satisfaction (DTSQs)^26^^,^^27^ questionnaires were completed before and after each diet phase and at last visit in the case of DTSQc.

### Endpoints

The primary endpoint was the mean glucose level during the last two weeks of each diet period. Secondary endpoints were the standard deviation (SD) of glucose levels, percent time above range (>10.0 mmol/L and >13.9 mmol/L), percent time in range (TIR) (3.9–10.0 mmol/L), weight, total cholesterol, LDL and HDL cholesterol, triglycerides, total daily insulin dose, DTSQc, DTSQs, and HCS total scores. Safety endpoints were percent time with low glucose levels <3.0 mmol/L and <3.9 mmol/L, number of severe hypoglycaemic events, blood ketone levels, and number of ketoacidosis events during each diet period.

Exploratory outcomes included changes in percent of time in tight range (3.9–7.8 mmol/L), percent of time in tighter range (3.5–7.8 mmol/L), Mean Amplitude of Glycemic Excursions (MAGE), coefficient of variation of glucose levels (CV), HbA1c, systolic and diastolic blood pressure, apolipoprotein A and B, apolipoprotein A/B ratio, daily mealtime insulin, daily basal insulin, total daily insulin dose to body weight ratio, as well as effects on glucose metrics during daytime (06:00–21:59 h) and nighttime (22:00–05:59 h).

### Safety assessments

Adverse events as well as the number of severe hypoglycaemic events (defined as unconsciousness due to hypoglycaemia, or requiring assistance), and occurrence of ketoacidosis were recorded at all contacts.

### Post-hoc analyses

The area under the curve (AUC) is viewed as an alternative method of calculating the mean glucose level. The AUC based on masked CGM data was compared between the traditional and moderate carbohydrate diet periods.

### Statistics

The Full Analysis Set (FAS) consists of all randomised participants with registered CGM data for at least one study period. Four different per protocol (PP) populations were also analysed (PP1-PP4) and defined at the clean-file meeting before the database was locked. PP1 includes individuals from the FAS with CGM measurements in both treatment phases that did not significantly deviate from the planned time period and with no protocol deviations regarding issues with diet compliance. PP2 comprises PP1 subjects with diet records confirming a difference in carbohydrate intake between treatment phases, featuring reduced carbohydrate intake during the moderate carbohydrate diet phase. PP3 includes PP2 subjects with a reduced percentage of energy intake from carbohydrates during the moderate carbohydrate diet phase, along with a relevant difference between treatment phases. PP4 includes PP1 subjects with at least 9 days of CGM data for at least 70% of the time in both diet periods. They are also shown in eTable 3.

The target sample size was set to 50 participants. Sample size calculations were performed for the Wilcoxon Singed Rank test under three different scenarios resulting in n = 54 to detect a mean difference in mean glucose levels of 1 mmol/L at an SD of 2.5 mmol/L, n = 48 for a mean difference = 1.5 and SD = 3.5, and n = 45 for a mean difference of 2.0 mmol/L and SD = 4.5. Additional conditions were 80% power, significance level α = 0.05, two-sided test. At the end, 54 subjects were recruited. The standard deviation was estimated using data from the GOLD trial.^28^

Descriptive data are presented as mean (SD), median (IQR), or range (minimum and maximum value) for continuous variables. Numbers and percentages are presented for categorical variables.

Statistical methods for crossover trials were applied using linear, log-linear, or generalised linear mixed effects models, as appropriate. Treatment (diet) and period were included as fixed effects and subjects as random effects. Normally distributed variables (CGM mean, CGM SD, weight, MAGE, blood pressure) were analysed using linear mixed effects models and log-normally (positive skewed) distributed variables (total cholesterol, LDL and HDL cholesterol, triglycerides, apolipoproteins, and ketone levels) using linear mixed effects after log-transformation. Other non-normally distributed numeric variables (time above range, time in range, time below range, insulin dose, DTSQs, DTSQc, and HCS) were analysed using linear mixed effects models with robust standard errors (HC3 method) to account for violations against distributional assumptions. For log-linear models, the regression coefficient of the treatment variable was exponentiated to obtain an estimate of the fold-change or relative risk between treatments. The plausibility of model assumptions was assessed visually by inspection of residual diagnostics, including residual plots and QQ-plots.

Statistical analyses were performed using SAS/STAT Software®, Version 9.4 of the SAS System for Windows (SAS Institute Inc., Cary, N.C.). All endpoints were evaluated on the FAS and PP populations (PP1–PP4). All statistical tests were two-sided and conducted at the 5% significance level. To account for multiple testing, a sequential testing procedure was employed. In case of a significant test for the primary endpoint, the entire probability mass α = 0.05 was transferred to the secondary endpoints in the order listed under *Endpoints* above until the first encounter of a non-significant test result. All those significant tests are considered confirmatory, and others are considered as exploratory findings.

The following changes from the protocol were made to the statistical analysis plan prior to database lock: the primary analysis method was changed from general linear models to mixed effects models. Mixed effects modelling allows for missing data in one of the diet periods and is generally considered the preferred approach to analysis of crossover trials. The full analysis set was re-defined accordingly from requiring CGM data in both diet periods to a more inclusive definition only requiring CGM data in one of the diet periods. Other minor changes included small modifications to statistical analysis methods for non-normal data to more powerful parametric mixed effects model approaches. Additional details may be found in the statistical analysis plan provided in eMethods3. The statistical analysis plan was signed before the database was locked.

### Role of the funding source

This study is supported by The Healthcare Board, Region Västra Götaland, The Dr P Håkansson Foundation and the Swedish state under the agreement between the Swedish government and the county councils, the ALF-agreement [ALFGBG-966173]. The sponsors did not have any role in the design and conduct of the study, collection, management, analysis and interpretation of the data and preparation, review, or approval of the manuscript.

## Results

### Participants

**Fig. 1:**
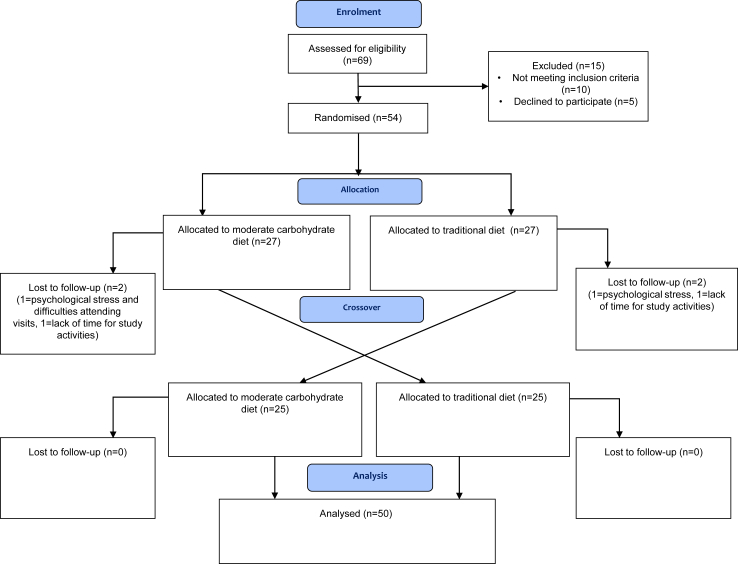
Flow chart over study participation according to CONSORT guidelines.

There were 69 individuals screened, 54 of them were randomised between March 2018 and March 2022. A flow diagram of participation according to CONSORT is shown in Fig. 1.

### Baseline characteristics

There were 50 individuals included in the FAS with a mean (SD) baseline HbA1c of 69 (SD 11) mmol/mol or 8.4% (SD 1.0) %, BMI 29 (SD 5) kg/m^2^, age 48 (SD 14) years, and 25 (50%) were female. All baseline data are shown in Table 1. Baseline characteristics were similar in the PP populations (eTable 4). The mean daily carbohydrate intake at inclusion was 40.1 (SD 6.9) percent of energy or 200 (SD 66) gram according to the diet records from the run-in period.

**Table 1:** Baseline characteristics of the study cohort.

Variable	Full Analysis Set (n = 50)
Age (years)	47.6 (14.1) 49.5 (22–73)
Female sex	25 (50%)
Diabetes duration (years)	25.4 (12.9) 25 (2–54)
Haemoglobin A1c (mmol/mol)	68.8 (10.8) 65 (58–105)
Haemoglobin A1c (%)	8.44 (0.99) 8.1 (7.5–11.8)
Weight (kg)	86.8 (17.3) 83 (60–130)
Body mass index (kg/m^2^)	29.2 (5.2) 28.9 (20.1–47.8)
Smoking	5 (10%)
CGM mean (mmol/L)^a^	9.2 (2.1) 9.0 (4.5–14.1)
Percent time in range 3.9–10 mmol/L^a^	53.1 (15.6) 49.4 (22.8–81.2)
Percent time above range >10 mmol/L^a^	38.7 (19.5) 37.7 (4.4–77.2)
Percent time in hyperglycaemia >13.9 mmol/L^a^	13.8 (11.8) 13.2 (0–46.1)
Percent time in hypoglycaemia <3.0 mmol/L^a^	3.33 (4.66) 1.62 (0–20.2)
Percent time below range <3.9 mmol/L^a^	8.2 (8.96) 5.1 (0–44.8)
Systolic blood pressure (mmHg)	134.1 (14.5) 133 (90–163)
Diastolic blood pressure (mmHg)	74.8 (9.6) 75.5 (55–103)
Total cholesterol (mmol/L)	4.14 (0.78) 4.2 (2.7–6.1)
Low-density lipoprotein (mmol/L)	2.31 (0.70) 2.1 (1–4.1)
High-density lipoprotein (mmol/L)	1.46 (0.43) 1.4 (0.7–2.6)
Triglycerides (mmol/L)	1.17 (0.62) 0.98 (0.4–2.8)
Insulin pump	17 (34%)
Total daily insulin dose (IU)^b^	58.6 (36.4) 47.5 (23.9–206)
Daily mealtime (bolus) insulin (IU)^b^	23.7 (17.9) 17.8 (5.8–91)
Daily basal insulin (IU)^b^	33.8 (21.3) 26 (11–136)
Total daily insulin dose (IU)^b^	58.6 (36.4) 47.5 (23.9–206)

Data are presented as mean (standard deviation), median (minimum–maximum value) for numeric variables and as numbers and percentages for categorical variables.

a CGM data available at baseline available for 36 individuals.

b Data on insulin available for 45 individuals.

### Primary endpoint

**Fig. 2:**
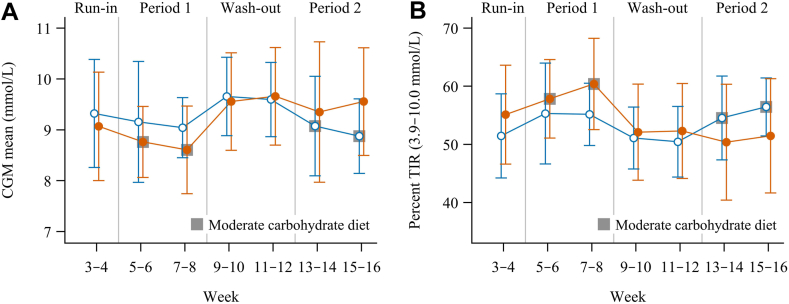
Mean glucose (A) and percent time in range, 3.9 –10.0 mmol/L (B) during the study period. Orange lines and orange filled markers: received moderate carbohydrate diet in the first period and traditional diet in the second period. Blue lines and white-filled markers: received traditional diet in the first period and moderate carbohydrate diet in the second period. Points and error bars represent means with 95% confidence intervals per two-week period.

Mean (SD) glucose level in the moderate carbohydrate and traditional diet estimated by masked CGM was 8.6 mmol/L (SD 1.7) and 9.2 mmol/L (SD 1.9), respectively, with a mean difference of −0.6 mmol/L (95% CI −0.9 to −0.3), p < 0.001 (Table 2, Fig. 2A).

**Table 2:** Evaluation of moderate carbohydrate diet vs traditional diet on primary and secondary endpoints in the FAS population.

Variable	Moderate carbohydrate diet	Traditional diet	Mean difference/Fold change (95% CI)
CGM mean (mmol/L)	8.6 (1.7)	9.2 (1.9)	−0.6 (−0.9 to −0.3) p <.001^a^
CGM SD (mmol/L)	3.6 (1.0)	3.7 (0.9)	−0.1 (−0.3 to 0.0) p = 0.15
Percent time in range 3.9–10 mmol/L	57.9 (13.4)	53.5 (16.9)	4.7 (1.3 to 8.0) p = 0.008
Percent time above range >10 mmol/L	32.8 (16.5)	38.4 (19.6)	−5.9 (−9.6 to −2.2) p = 0.003
Percent time in hyperglycaemia >13.9 mmol/L	10.4 (10.0)	13.7 (13.1)	−3.6 (−6.1 to −1.1) p = 0.006
Percent time in hypoglycaemia <3.0 mmol/L	3.8 (4.1)	3.2 (3.3)	0.6 (−0.4 to 1.6) p = 0.26
Percent time below range <3.9 mmol/L	9.4 (7.3)	8.1 (6.1)	1.2 (−0.4 to 2.9) p = 0.13
Weight (kg)	86.7 (17.4)	86.3 (17.7)	0.0 (−0.4 to 0.4) p = 0.98
Total cholesterol (mmol/L)	4.1 (3.6–4.5)	4.2 (3.6–4.8)	0.98 (0.95 to 1.01)^b^ p = 0.28
Low-density lipoprotein (mmol/L)	2.2 (1.7–2.7)	2.2 (1.7–2.6)	0.98 (0.93 to 1.03)^b^ p = 0.44
High-density lipoprotein (mmol/L)	1.4 (1.1–1.8)	1.5 (1.2–1.7)	0.99 (0.95 to 1.04)^b^ p = 0.68
Triglycerides (mmol/L)	0.9 (0.6–1.3)	1.0 (0.7–1.3)	0.99 (0.88 to 1.10)^b^ p = 0.79
Total daily insulin dose (IU)	56.2 (39.6)	59.7 (39.1)	−3.3 (−7.5 to 0.9) p = 0.12
DTSQs total score	28.7 (5.4)	27.4 (6.1)	1.4 (0.2 to 2.5) p = 0.026
Hypoglycaemia confidence scale mean score	3.5 (0.5)	3.5 (0.4)	0.0 (−0.1 to 0.1) p = 0.55

Data are presented as mean (SD) and mean difference (95% CI) or median (IQR) and fold-change (95% CI).

Statistical analyses were performed using linear mixed effects models with treatment (diet) and period as fixed effects and subject as random effect.

Abbreviations: CI, confidence interval; IQR, interquartile range; SD, standard deviation.

a This is a confirmatory finding according to the pre-defined testing procedure (see Statistical Analysis Plan, eMethods3, for details).

b Fold change between groups is presented.

In the PP analyses where food records showed a relevant difference between diet phases in either grams of carbohydrates (PP2, n = 27) or percent of energy (PP3, n = 20), comparable results were seen. The difference was −0.7 mmol/L (95% CI −1.15 to −0.17), p = 0.01, and −0.6 mmol/L (95% CI −1.19 to 0.07), p = 0.08, respectively. PP1 and PP4 showed comparable results (eTable 5).

### Secondary endpoints

There was no significant difference in SD of glucose values between diet phases (Table 2). Time in range increased in the moderate carbohydrate phase by 4.7% (68 min/24 h) (95% CI 1.3 to 8.0), p = 0.008 (Fig. 2B). There were 8 (16%) of individuals who reached the target of TIR of 70% during moderately reduced carbohydrate diet and 4 (8%) during the traditional diet. Time above range (>10 mmol/L) decreased by 5.9% (85 min/24 h) (95% CI −9.6 to −2.2, p = 0.003), compared to traditional diet, and time above range (>13.9 mmol/L) decreased by 3.6% (52 min/24 h) (95% CI −6.6 to −1.1, p = 0.006). DTSQs total score increased by 1.4 points, 95% CI 0.2 to 2.5, p = 0.026 (Table 2).

There were no significant differences in weight, total, LDL or HDL cholesterol, triglycerides, total insulin dose, DTSQc, or hypoglycaemia confidence scale between the two diet phases (Table 2).

Results from the PP populations were all consistent with the FAS analyses. Analyses of all PP populations of TIR showed consistent patterns with increases in TIR ranging from 3.5 to 5.1% and decreases in time above range (>10 mmol/L) ranging from −5.5 to −6.6, and time above range (>13.9) between −2.6 and −3.2 (eTable 5).

### Safety endpoints

No significant differences in hypoglycaemia were shown between moderate carbohydrate and traditional diet in the range <3.9 mmol/L: 1.2% (95% CI −0.4 to 2.9, p = 0.13) or in the range <3.0 mmol/L: 0.6% (95% CI −0.4 to 1.6, p = 0.26) (Table 2) in the FAS population, nor were there any cases of severe hypoglycaemia.

**Fig. 3:**
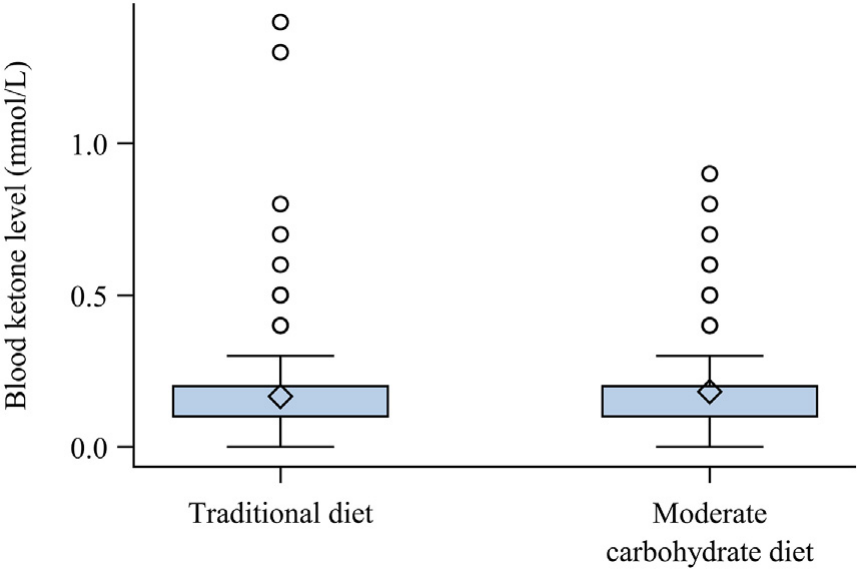
Distribution of blood ketone levels during traditional diet and moderate carbohydrate diet. The box represents the lower and upper quartile. The diamond is the mean. Points represent individual values (outliers) above the upper quartile plus 1.5 times the interquartile range. The whiskers are the smallest and largest value, outliers excluded. The number of blood ketone samples was n = 413 during traditional diet and n = 467 during moderate carbohydrate diet.

The mean ketone level was 0.17 mmol/L (SD 0.14) during traditional diet and 0.18 mmol/L (SD 0.13) during moderate carbohydrate diet (fold-change 1.09, 95% CI 1.01–1.18, p = 0.02) (Fig. 3). Ketone levels remained low during the entire study period during both diet phases. The maximum ketone level measured was 1.4 mmol/L during traditional diet and 0.9 mmol/L during moderate carbohydrate diet. There were no cases of ketoacidosis during the study.

### Exploratory endpoints

Percent of time in tight range (3.9–7.8 mmol/L) increased in the moderate carbohydrate diet phase by 4.5% (95% CI 1.2–7.8), p = 0.008, and percent of time in target range (3.5–7.8 mmol/L) increased by 4.8% (95% CI 1.2–8.4), p = 0.01, compared to the traditional diet phase (eTable 6).

There were no significant differences in glucose variation metrics MAGE or CV measured by masked CGM, systolic or diastolic blood pressure, ApoA, ApoB, ApoA/ApoB ratio, daily mealtime (bolus) insulin doses, daily basal insulin doses, or daily insulin dose to bodyweight ratio (IU/kg) between the two diet phases.

In the PP populations most changes were of similar magnitude, although total daily insulin dose to bodyweight ratio was slightly lower in the moderate carbohydrate diet phase, and the difference was statistically significant in all PP populations (eTable 7).

### Difference between diets in CGM endpoints during daytime and nighttime

Differences in CGM endpoints between diets were similar in magnitude. Significant differences existed during daytime and were similar in direction although weaker and not significant during nighttime (eTable 8).

### Macronutrient intake

Of the 50 individuals included in FAS analyses, 47 individuals had diet records in at least one diet phase and 39 individuals in both phases. From these individuals, the mean (SD) total amount of carbohydrates in percent of energy was 34 (SD 6) and 41 (SD 7) percent, and 155 (SD 40) and 187 (SD 51) grams in the moderate carbohydrate and traditional diet phase, respectively. Total amount of fat and protein in percent of energy was 43 (SD 7) and 37 (SD 7), and 18 (SD 3), and 17 (SD 3) in the moderate carbohydrate and traditional diet, respectively. In the PP populations the difference in carbohydrate intake between the diet phases ranged from 55 to 59 g, or 8 to 11 percent of energy from carbohydrates (eTable 9).

### Adverse events

There were neither any serious adverse events nor severe adverse events reported during the study. All adverse events are shown in eTable 10 and eTable 11. Medical history is summarised according to ICD-10 and described in eTable12. Prior and concomitant medications were summarised by higher level anatomical therapeutic classification (ATC) group and described in eTable 13 and eTable 14.

### Post-hoc analyses

The mean glucose measured by the area under the curve (AUC) of glucose levels by CGM was 0.7 mmol/L lower (95% CI 0.3–1.0 mmol/L, p < 0.001) during moderate carbohydrate diet compared to traditional diet. Results of AUC for the PP populations showed similar patterns favouring effects on AUC of the moderate carbohydrate diet (eTable 15). For PP2 where all participants differed in their amounts of carbohydrates between the two treatment phases the difference in AUC was 0.8 mmol/L (95% 0.2–1.3 mmol/L, p = 0.015) in favour of the moderate carbohydrate diet.

## Discussion

A moderate carbohydrate diet leads to significantly improved glucose control compared to a traditional diet with higher carbohydrate content in adults with T1D. The primary outcome, mean glucose level, was significantly reduced. Other endpoints showed more time in range, time in tighter ranges, and less time with high and very high glucose levels as well as increased treatment satisfaction. There was no increase in time in hypoglycaemia or cardiovascular risk factors such as lipids and blood pressure, no ketoacidosis, severe elevated ketone levels, or adverse events during the moderate carbohydrate diet.

The lack of diet studies in T1D may be due to need for them to be driven academically, little interest from the pharmaceutical industry, and their time-consuming nature. A few earlier studies showed improvements in glucose control with low carbohydrate diets^18^, ^19^, ^20^, ^21^, ^22^ in adults with T1D, but there have also been concerns about safety regarding the risk of elevated lipids and increased risk of ketoacidosis and hypoglycaemia.^14^^,^^23^ Because evidence regarding efficacy and safety are lacking and the possible increased risk of ketoacidosis and severe hypoglycaemia, low carbohydrate diets are not yet recommended in dietary guidelines for people with T1D.^2^ Earlier studies were either not randomised or very small. Low carbohydrate diets can differ widely in recommended carbohydrate amount, and most studies were of very low carbohydrate intake (less than 120 g and 5% of energy).^18^, ^19^, ^20^, ^21^, ^22^, ^23^ In this study our goal was to ensure a healthy diet to increase adherence for longer time periods. Safety was also a priority; therefore, we used a moderate carbohydrate diet with 30% of energy from carbohydrates compared to the traditional 50% which would make a clinically relevant difference in carbohydrate amount, but still likely be safe in both the short- and long-term. It also included healthy foods such as wholegrains, legumes, vegetables, and unsaturated fats according to guidelines^1^, ^2^, ^3^ to avoid negative health effects such as elevated blood pressure or dyslipidaemia.

The obtained effect on mean glucose corresponds to approximately 3 mmol/mol (0.3%) reduction in HbA1c^29^ which has been associated with a reduced risk of retinopathy.^30^ Mean glucose level was chosen as the primary endpoint since the true glucose level without increased time in hypoglycaemia is a marker of both acute and long-term complications. Moreover, an increase in time in range of 5%, comparable to the current results of 4.7%, is considered a clinically relevant difference according to CGM guidelines.^31^ Although participants increased in time in range the majority did not reach targets of 70 percent TIR indicating the need of additional improvements in diabetes care for many persons to achieve targets of glucose control. Further positive effects were seen in the decrease in time above range (>10 mmol/L) by 5.9% as well as the decrease in time above range (>13.9 mmol/L) of 3.6%. Since an exponential relationship exists between glucose levels and diabetes complications it is important to reduce time with very high glucose levels for all individuals with T1D.^32^

One possible mechanism for the decrease in mean glucose level as well as the other positive effects on glucose control during the moderate carbohydrate diet may be due to fewer glucose excursions and peaks. Predicting the precise amount of insulin required becomes challenging due to several factors influencing insulin uptake such as physical activity during the day. Consequently, reducing the carbohydrate intake per meal potentially mitigates glycaemic peaks even if the insulin dosage is not entirely correct.

The current results indicate that a moderate carbohydrate diet should be included in dietary guidelines for persons with T1D as an alternative for decreasing mean glucose levels and that it can be considered safe, although it is important that it is possible for individuals to receive dietary advice from a dietitian to make sure the diet is healthy in terms of fat and carbohydrate sources.

Strengths of the study include randomised crossover design which reduces risk of confounding factors and person-to-person variation. It also included a detailed individual diet plan for each participant and during each diet phase with regular follow-up to increase adherence. Finally, it included a structured insulin management plan to keep it as comparable as possible between diet phases.

In this study participants measured ketone levels twice a week in the morning and evening, and they were asked to report adverse events during follow-up to determine risk of ketoacidosis and other possible negative effects of the diet. During moderate carbohydrate diet, ketone levels were slightly elevated, although levels overall were low and never were severely elevated. There were no cases of ketoacidosis, indicating that this level of carbohydrate intake may be safe. Insulin to body weight ratio was slightly decreased in the moderate carbohydrate diet, indicating that insulin doses were decreasing along with carbohydrate intake, which logically would be expected.

The total study period of 12 weeks and diet phases of 4 weeks each was intentional making it possible for participants to comply with dietary changes and all study procedures including keeping food diaries, measuring ketone levels, and using masked CGM to provide detailed glucose and diet data. This study mainly elucidates physiological effects and effects on glucose control of a moderate carbohydrate diet, and individual preferences likely exist which should be considered in clinical practice in order to increase compliance during longer treatment periods.

The food diaries indicated that differences in carbohydrate intake between the phases were less than planned, and a minority of participants lacked food records. Furthermore, carbohydrate intake was self-reported and thus may be biased.^33^^,^^34^ Although food diaries were not registered in both phases by all participants the majority had recordings and the per protocol analyses using these data confirmed effects on glucose control for individuals differing in carbohydrate intake. Of note is that only adults with HbA1c of ≥58 mmol/mol (7.5%) were included in the study which may restrict results to this population.

Although the primary endpoint, mean glucose level, can be viewed as confirmatory, other glucometrics should be viewed as exploratory and preferentially confirmed in other studies. However, since there is a strong correlation between mean glucose level, time in range, and time in hyperglycaemia, our findings further support likely beneficial effect on other important glucometrics.^35^^,^^36^

In summary, this study shows that in persons with T1D a moderate carbohydrate diet is more effective than a traditional diet with a higher amount of carbohydrates in terms of decreasing mean glucose levels and time above range and increasing time in range and treatment satisfaction without increased risk of hypoglycaemia, dyslipidaemia, or ketoacidosis. These results show that a healthy moderate carbohydrate diet can be considered as a safe and effective treatment option for individuals with T1D which extends possibilities for more differentiated diabetes care and provides further options to individualising diet treatment.

## Contributors

SS, AFÓ, and ML designed the trial. ML, ET, UR, SH were the principal investigators on the trial sites, SS and SI were dietitians, and AFÓ and ME were diabetes nurses in the trial. HI was responsible for the statistical analyses. SS drafted the manuscript. ML had the overall responsibility of the trial. All authors have critically reviewed and approved the final manuscript.

## Data sharing statement

Data from this study can be made available from the corresponding author upon reasonable request.

## Declaration of interests

AFÓ has received lecture fees from Nordic Infucare, and ML has received research grants from Eli Lilly, Novo Nordisk and been a consultant or received honoraria from Astra Zeneca, Eli Lilly, Novo Nordisk, and Nordic Infucare all outside the current study. SH has lectured for Novo Nordisk. ET, HI, ME, SI, SS and UR have nothing to declare.

## Appendix A Supplementary data

Supplementary Material 2

eTables 1–15
