# Supplementary material for: The effect of carbohydrate intake on glycaemic control in individuals with type 1 diabetes: a randomised, open-label, crossover trial
Source: Lancet Reg Health Eur. 2023 Dec 19;37:100799. doi: 10.1016/j.lanepe.2023.100799 (PMC10866914; doi:10.1016/j.lanepe.2023.100799)
Supplement: Supplementary Material 1 [file mmc1.pdf]

1 Title Page

**Protocol Title:**	**The effect of carbohydrate content in the diet on mean glucose levels in type 1 diabetes**
Sponsor:	NU-Hospital Group, Trollhättan and Uddevalla, Sweden
CGM System:	DexComG4 (DexCom Corporation)
Protocol Release date:	2017-05-22
GCP Statement:	This study will be performed in full compliance with ICH and all applicable local Good Clinical Practices (GCP) and regulations. All required study documentation will be archived as required by competent authorities.

Marcus Lind
4 Sept 2018
MD, PhD

2 Synopsis

A keystone in preventing diabetic complications in patients with type 1 diabetes is good glycaemic control. In the clinical practice we have noticed that many patients finds it a lot easier to keep their blood sugar levels stabile, with less fluctuations, if their intake of carbohydrates in the diet is lower. There is on the other hand probably an increased risk of ketoacidosis at a very low intake of carbohydrates. Therefore, we think that it could be a risk for the patients to eat a low carb-high fat diet (LCHF), which is extremely low in carbohydrates. There is a lack in the literature of studies that evaluates the effect of a moderate intake of carbohydrates, and the pros and cons with this diet for patients with type 1 diabetes.

The current trial is a cross-over design and 12 weeks in duration, where patients will be randomized to; 1) a diet with moderate carbohydrate content for 4 weeks and 2) traditional diabetic diet with low GI for 4 weeks and a wash-out period for 4 weeks. The primary endpoint is the effect on mean glucose levels measured with blinded CGM. There will be 50 subjects included in the study.

3 List of Abbreviations and Definitions of Terms

AE = Adverse event

BMI = Body mass index

CGM = Continuous glucose monitoring

DTSQ=Diabetes Treatment Satisfaction Questionnaire

FPG = Fasting plasma glucose

HbA1c = Glycated haemoglobin

PG = Plasma glucose

SAE = Serious adverse event

SD = Standard deviation

SMBG = Self-Monitoring Blood Glucose

T1DM = Type 1 Diabetes Mellitus

Table of Contents

1 Title Page ... 1
2 Synopsis ... 2
3 List of Abbreviations and Definitions of Terms ... 2
4 Study conduct & Oversight ... 5
4.1 Sponsor and Principal Investigator (PI) ... 5
4.2 Diet interventions ... 5
4.3 CGM-system ... 5
4.4 Executive committee ... 5
4.5 Lab ... 5
4.6 Investigator and site ... 5
4.7 Data Management & Statistics ... 5
5 Introduction & Background Information ... 6
5.1 Background and survey of the field ... 6
5.2 Study Population ... 6
5.3 Purpose/aim of the study ... 6
5.4 Primary Objective ... 7
5.5 Secondary Objectives ... 7
6 Importance ... 7
7 Trial Design ... 7
7.1 Design ... 7
7.2 Treatments ... 7
7.3 Randomization ... 7
7.4 Duration ... 7
7.5 Endpoints ... 8
7.5.1 Primary endpoint ... 8
7.5.2 Secondary endpoints ... 8
8 Selection of subjects ... 9
8.1 Inclusion and exclusion criteria ... 9
8.1.1 Inclusion criteria ... 9
8.1.2 Exclusion criteria ... 9
9 Treatment of Subjects ... 10
9.1 Treatment procedures ... 10
9.2 Rescue Criteria ... 11
9.3 Procedures for monitoring subject compliance ... 11
9.4 Treatment Satisfaction and other questionnaires ... 11
10 Trial Procedures ... 12
10.1 Screening and information ... 13
10.2 Visit 1 – Information and inclusion (week 1-4) ... 13
10.3 Visit 2 – Run-in period (week 1-4) ... 13
10.4 Visit 3 – Randomization and start of diet 1 (week 5) ... 13
10.5 Follow-up (telephone) the day after intervention start, week 5 ... 14
10.6 Follow-up (telephone) 4 days after intervention start, week 5 ... 14
10.7 Follow-up, 7 days after intervention start (telephone), week 6 ... 14
10.8 Follow-up, 14 days after intervention start, week 7 ... 14
10.9 Visit 4 – Intervention 1 finished, week 8 ... 14
10.10 Wash-out period, telephone contact at week 11 ... 15
10.11 Visit 5 – Intervention 2 start, week 13 ... 15
10.12 Follow-up (telephone) the day after intervention start, week 13 ... 15

10.13 Follow-up (telephone) 4 days after intervention start, week 13 15
10.14 Follow-up, 7 days after intervention start (telephone), week 14 15
10.15 Follow-up, 14 days after intervention start, week 15.......... 16
10.16 Visit 6 – Intervention 2 finished, week 16 16
11 Asessment of Safety.......... 16
11.1 Hypoglycaemia, ketons, ketoacidosis and gastrointestinal symptoms 16
11.2 Adverse Events (AE) 16
11.2.1 Definition of AE 16
11.2.2 Hypoglycaemias.......... 17
11.2.3 Reporting of Adverse Events (AE) 17
11.3 Serious Adverse Events (SAE) 17
11.3.1 Definition of SAE 17
11.3.2 Reporting of SAE.......... 18
12 Statistics 18
12.1 Populations.......... 18
12.1.1 Full analysis set (ITT-population) 18
12.1.2 Per-Protocol population 18
12.1.3 Safety Population 18
12.1.4 Sensitivity analysis.......... 18
12.2 General Statistical Methodology.......... 18
12.3 Efficacy analyses.......... 19
12.3.1 Primary efficacy analysis.......... 19
12.3.2 Secondary efficacy analyses 19
12.4 Statistical Analysis Plan.......... 19
12.5 Sample Size Calculation 19
12.6 Safety analyses.......... 20
13 Premature termination of the trial 20
14 Data Handling and Record Keeping 20
14.1 Data Collection 20
14.2 Data Management 20
14.2.1 Study Database & Data Entry 20
14.2.2 Clean File & Database Lock.......... 20
14.3 Data Retention & Archiving 20
15 Access to Source Data/Documents 21
16 Ethics.......... 21
17 Insurance 21
18 Compensation 21
19 Publication Policy 21
20 References.......... 21

4 Study conduct & Oversight

4.1 Sponsor and Principal Investigator (PI)

This is an investigator-initiated trial. The sponsor is NU-Hospital Group, Trollhättan and Uddevalla, Sweden. Marcus Lind is the PI of the study.

4.2 Diet interventions

The two intervention diets: 1) a diet wit moderate amounts of carbohydrates and 2) a traditional diabetic diet with a higher carbohydrate content, will be planned and individualized by a registered dietitian in accordance with current dietary guidelines.

4.3 CGM-system

DexCom Corporation will provide the DexCom G4 systems (CGM) and sensors during the trial.

4.4 Executive committee

Marcus Lind, MD, PhD
Department of Molecular and Clinical Medicine, Sahlgrenska Academy, University of Gothenburg, Gothenburg, Sweden and NU-Hospital Organization, Uddevalla, Sweden

Sofia Isaksson, RD, PhD-student
Department of Molecular and Clinical Medicine, Sahlgrenska Academy, University of Gothenburg, Gothenburg, Sweden and NU-Hospital Organization, Uddevalla, Sweden

Finna Arndis Olafsdottir, diabetes nurse
NU-Hospital Organization, Uddevalla, Sweden

4.5 Lab

Laboratory samples will be measured at the site, at the NU-Hospital Group, Uddevalla, Hospital, Uddevalla, Sweden.

4.6 Investigator and site

Marcus Lind, MD, PhD, NU-hospital Organization, Uddevalla, Sweden. Uddevalla Hospital will be the place were the study will be conducted.

4.7 Data Management & Statistics

Data Management (including the randomization system) and statistical analysis are planned to be performed by Statistiska Konsultgruppen, Gothenburg, Sweden. The use of a eCRF system is also planned for the study.

5 Introduction & Background Information

5.1 Background and survey of the field

A keystone in preventing diabetic complications in patients with type 1 diabetes (T1DM) is good glycaemic control. Good glycemic control is related to a decreased risk in developing complications to T1DM and also with a lower mortality (1-3). Today, mortality is still much higher in patients with type 1 diabetes compared to the rest of the population and this could probably decrease a lot if more patients could reach their targets for blood sugar levels (3, 4).

In the clinical practice we have noticed that many patients finds it a lot easier to keep their blood sugar levels stabile, with less fluctuations, if their intake of carbohydrates in the diet is lower. There is a certain logic to this because if a insulin dose is too small, like for example if the insulin dose is incorrect by 20 %, a greater rise in blood sugar will occure in the case of higher amont of carbohydrate in the meal (5). It is also known that even during standardised conditions it is hard to predict an exact insulin dose and therefore it is hard for patients to make an exact insulin dose to the amount of carbohydrates even if they try to optimise the dose (5). This is due to the fact that there is a intraindividual variation in the uptake of insulin in the body of all availiable insulin sorts. There is also many other things that has an effect like physical activity during the past 24 hours, stress, infections, inflammation and distress and it is therefore hard to standardise (5).

There is on the other hand probably an increased risk of ketoacidosis at a very low intake of carbohydrates. This is because the patients decreases their insulindoses and lack of insulin produces ketones (5). Because of this we think that it could be a risk for the patients to eat a very low carb-high fat diet (LCHF), wich is extremely low in carbohydrates.

There is a lack of randomized trials addressing whether a healthy diet with less carbohydrates leads to a better glycaemic control than a diet with a greater amount of carbohydrates. Evidence as a basis for guidelines is hence overall lacking and today only general guidelines exist, mainly based on studies in persons with type 2 diabetes or healthy individuals (6, 7). However, type 2 diabetes is a totally different disease; e.g. most patients do not need exogenous insulin and almost all still have their own insulin production (5). To our knowledge, only a few, small studies have been performed examining the effect of a low carbohydrate diet on people with T1DM. Although they show promising results on HbA1c (8, 9), they are either not randomised (8) or very small (9) and have therefore not generated enough scientific evidence to know whether the results can be translated to people with T1DM in general.

The current trial is of cross-over design and 12 weeks in duration, where patients will be randomized to; 1) a diet with moderate carbohydrate content for 4 weeks and 2) traditional diabetic diet with low GI for 4 weeks and a wash-out period for 4 weeks. The primary endpoint is the effect on mean glucose levels measured by CGM.

5.2 Study Population

Adult patients in Sweden with type 1 diabetes with a HbA1c ≥ 58 mmol/mol.

5.3 Purpose/aim of the study

The aim of this study is to analyse the effect of a diet with a moderate amount of carbohydrates (30 E %) and compare it with a traditional diabetic diet with a higher content of carbohydrates (50 E %) on mean glucose level, high and low glucose levels , and the risk of ketoacidocis in patients with type 1 diabetes.

5.4 Primary Objective

The primary objective is to examine whether a diet with a moderate amount of carbohydrates is more effective than a traditional diabetic diet with a higher amount of carbohydrates on *mean glucose levels (measured by CGM)* in patients with type 1 diabetes.

5.5 Secondary Objectives

Secondary objectives are to examine whether a diet with a moderate amount of carbohydrates has a different effect than a traditional diabetic diet with a higher amount of carbohydrates on standard deviation of blood glucose levels, MAGE, time in hypoglycaemia, time in hyperglycemia, time in euglycemia, hypoglycaemia confidence, treatment satisfaction, HbA1c, blood lipids, weight, total insulin dose and ketones in patients with type 1 diabetes.

6 Importance

It is of great importance to gain knowledge of the potential benefits or risks associated with a lower amount of carbohydrates in the diet in type 1 diabetes. If it has beneficial effects on the mean glucose level without an elevated risk of hypoglycaemia or ketoacidosis it will be an optional treatment for people with T1DM that have unsatisfactory blood glucose levels. If it on the other hand doesn´t show any beneficial effects it will be important knowledge in the healthcare when educating patients about diet. This study can therefore be of importance for upcoming diabetes nutrition recommendations.

7 Trial Design

7.1 Design

A randomized, non-blinded, cross-over clinical trial.

7.2 Treatments

The interventions will be a diet with a moderate amount of carbohydrates (30 E %) which will be compared to a traditional diabetic diet with a higher content of carbohydrates (50 E %). All subjects will be wearing blinded CGM to measure blood sugar levels during the study. Baseline data of blood sugar levels and diet composition will also be measured.

7.3 Randomization

Consenting patients will be randomized to a diet with a moderate amount of carbohydrates or traditional diabetic diet for 4 weeks, with an intermittent wash-out period for 4 weeks and then have the other intervention diet for 4 weeks.

Patients will be initially randomized 1:1, to either of the groups. A centralised web system (handled by Statistiska Konsultgruppen) will be used for randomisation. Each patient will be assigned a unique and anonymous Subject ID at randomisation.

DexCom Corporation will provide the DexCom G4 systems (CGM) and sensors.

7.4 Duration

The expected study duration for each participant is 16 weeks, including an assumed mean run-in period of 4 weeks.

7.5 Endpoints

7.5.1 Primary endpoint

The primary endpoint is the difference in mean glucose level (measured by CGM) between the two different diet periods (the last 2 weeks of each diet period).

7.5.2 Secondary endpoints

Secondary endpoints are the following:

Clinical variables:

The difference in standard deviation of glucose levels (measured by CGM during last two weeks) between the two different diet periods.

The difference in the proportion of time with high glucose levels (measured by CGM during last two weeks) between the two different diet periods. (above 10.0 mmol/l and above 13.9 mmol/l respectively)

The difference in the proportion of time with euglycaemic levels (5.5-10.0 mmol/l and 3.9-10.0 mmol/l respectively) (measured by CGM during last two weeks) between the two different diet periods.

The difference in weight between the two different diet periods.

The difference in total cholesterol between the two different diet periods.

The difference in LDL cholesterol between the two different diet periods.

The difference in HDL cholesterol between the two different diet periods.

The difference in triglycerides between the two different diet periods.

The difference in total insulin dose between the two different diet periods.

Treatment satisfaction, physical activity and hypoglycaemia confidence:

The difference in DTSQc score at the end of the study (week 16) between the two study periods.

The difference in DTSQs scores between the two different diet periods.

The difference in hypoglycaemia confidence scores between the two different diets

Other Exloratory endpoints

The difference in MAGE (measured by CGM during two weeks) between the two different diet periods.

The difference in HbA1c between the two different diet periods.

The difference in apolipoproteins between the two different diet periods.

Safety Endpoints

The difference in the proportion of time with low glucose levels (measured by CGM during two weeks) between the two different diet periods (below 3.0 mmol/l and below 3.9 mmol/l respectively).

The difference in the mean number of severe hypoglycaemic events between the two diet periods defined as unconsciousness due to hypoglycaemia or need of assistance from another person to resolve the hypoglycaemia

The difference in keton levels between the two diet periods.

Occurance of ketoacidosis during the study period and differences between the two diet periods.

8 Selection of subjects

Patients fulfilling all inclusion and no exclusion criteria will have their HbA1c levels analysed with capillary measurement at the Uddevalla hospital. The study is planned to include 50 patients randomized 1:1, to a diet with moderate carbohydrate content or a traditional diabetic diet with higher carbohydrate content . Treatment period will be 4 weeks for each diet period, with a wash-out period of 4 weeks between treatments.

8.1 Inclusion and exclusion criteria

8.1.1 Inclusion criteria

1. Type 1 diabetes
2. Adults 18 years or older
3. Written Informed Consent
4. HbA1c ≥ 58 mmol/mol (7,5 % DCCT standard)

8.1.2 Exclusion criteria

1. Pregnancy or planned pregnancy for the study duration
2. Severe cognitive dysfunction or other disease, which is judged by the physician to be not suitable for inclusion.
3. Problems with compliance to the intervention diets (excluding a lot of the foods that are common in the diets like wholegrain/beans/lenthils/fruit/vegetables) because of personal preferences, other diseases, stomach problems etc.
4. Diabetes duration < 1 year
5. Planned change in diabetes treatment (eg. Start with insulin pump, CGM etc.) during the study period.
6. Other investigator-determined criteria unsuitable for patient participation.
7. Continuous use of paracetamol use where not other pain killers can be used (because paracetamol use can affect the accuracy of the Dexcom CGM system).

9 Treatment of Subjects

A web randomization system will be used to allocate the subjects their diets groups.

9.1 Treatment procedures

All subjects in the trial will be instructed regarding basic information on insulin dosing, such as bolus correction, types of food elevating glucose levels and the effect of physical activity on glucose control. This information will be provided at the same level as in clinical practice for patients with type 1 diabetes, i.e. to guarantee that all subjects have basic skills for dosing insulin before the interventions begins.

All patients will wear a sensor and a blinded CGM system during a run-in period and during 2 weeks of the intervention periods, and during 2 weeks of the wash-out period. In total the will use the CGM system for 8 weeks. Care-givers will down-load data from the CGM system at all clinical visits. At clinical visits and at telephone contacts the research staff will discuss the current diet and try to motivate the patient to keep the diet in accordance to the protocol. Further, the research staff will perform a general check that the patient takes a reasonable amount of basal insulin, both from a safety perspective and efficacy perspective. In accordance the research staff will check if the patient performs adequate adjustments of prandial insulin doses and adjust these if needed.

Regarding the subjects that are eating the diet low in carbohydrates the insulin regimen needs to be adjusted when the subjects start with the diet. They will probably need to lower their insulin doses by about 20-30 % compared to when they are eating normal amounts of carbohydrates in the diet, but this will be calculated individually depending on their regular diet as well as other affecting factors. And it is also possible that the lowering of insulin doses is just initial, and that they will need to use higher doses again after the first days of the new diet.

At clinical visits the care-giver will discuss glucose levels measured by SMBG with the patient if the glucose levels is unsatisfying. All patients will have the possibility to contact the responsible staff for the trial for additional support between the visits if needed, e.g. technical problems with the CGM-system, the diet or insulin dosage but extra visits will not be planned with the aim of improving the glycaemic control. Patients will be motivated in measuring blood glucose levels in accordance with guidelines, i.e. at least 4 times a day. At the visits before each treatment period patients will be checked for general skills adopted on dosing insulin, types of foods that elevate glucose levels and the influence of physical activity on glucose levels.

HbA1c will be measured at baselineand at the starting point of each treatment period and in the end of each diet period. Blood lipids (total cholesterol, LDL-, HDL, apolipoproteins, triglycerides) will be measured at baseline and before and after each treatment period. Blood pressure and weight will also be measured at baseline and before and after each treatment period. Length will be measured at baseline.

All subjects will receive an individualised diet plan before the start of the diet interventions. This will include recipies, examples of meals with the correct carbohydrate content, examples of a whole day and ideas for suitable foods and meals that fits with the diet plan as well as pedagogical examples of how much carbohydrates they can eat during the day. They will also have the chance to discuss this with the study dietitian and to individualize the diet according to their needs and preferences.

We will also ask and register concomitant medications at every visit including; types of insulins, insulin doses, other glucose lowering agents, , blood lipid lowering medications, antihypertensives and paracetamol.

9.2 Rescue Criteria

If the clinician or diabetic educator determines that the "moderate carbohydrate diet" is associated with severe risks, e.g., severe hypoglycaemia, the treatment shall be stopped and the patient will receive the traditional diet instead.

9.3 Procedures for monitoring subject compliance.

Protocol compliance and adherence will be checked at each patient contact. The dietitian will ask about the diet and the diabetes nurse/doctor about the blood glucose levels and insulin dosage. Adherence to the diets will also be checked by recording the food intake during 4 days during each intervention period. The food records will afterwards be calculated regarding macronutrients and energy intake by the dietitian.

9.4 Treatment Satisfaction and other questionnaires

The Diabetes Treatment Satisfaction Questionnaire, DTSQ has been used in many diabetes therapy clinical trials and is a validated questionnaire consisting of 8 questions. Two versions are used, the DTSQs and DTSQc, where the DTSQs is used for recording the current treatment satisfaction and the DTSQc for patients to retrospectively compare the two treatments. The subjects will fill in the questionnaire DTSQs at baseline and before and after each diet period and the DTSQc after the last diet period.

The hypoglycaemia confidence questionnaire is a 9-item scale that evaluates patient confidence regarding their ability to prevent and address hypoglycemic events. The subjects will fill in the questionnaire at baseline and before and after the diet periods.

The questionnaires will be completed at the study site. The patients will be allowed to individually complete the questionnaires in a reasonably quiet environment . It will be emphasized that patients complete the questionnaires prior to clinical measurements and before meeting a dietistian/nurse/doctor. Questionnaires should be answered by the patient alone; however, the study personel will be informed to help patients complete the questionnaires, if necessary, but without influencing patients' responses. Only the anonymous Subject ID will be used to identify questionnaires to ensure patient confidentiality. Study personel should check questionnaires for completeness. The PI shall ensure that appropriate study training is provided.

Physical activity will be measured with a questionnaire at baseline and before and after each diet period. The purpose of measuring this is to be able to measure the total energy expenditure to calculate energy requirements to plan and individualize the study diets. The measurements after the study periods is to check that physical activity haven't changed during the study, because in that case this could influence the blood glucose levels in different ways.
We will use validated questions and scales to measure physical activity level and changes.

10 Trial Procedures

Trial procedure during the run-in phase and trial is schematically shown below:

Week	1 - 4		5	6	7	8	9	10	11	12	13	14	15	16
Visit	Run-in phase													
Visists at site	1	2	3			4					5			6
Screening/ Information	X													
Informed consent	X													
Randomization		X												
Blinded CGM		X	X		X	X			X	X			X	X
Demographics, medical history	X													
HbA1c		X	X			X					X			X
blood lipids, apolipoproteins		X	X			X					X			X
Blood pressure		X	X			X					X			X
BMI		X	X			X					X			X
Diet and insulin record		X				X								X
DTSQs, Hypoclycemia confidence, Physical activity		X	X			X					X			X
DTSQc														X
Insulin dose		X	X			X					X			X
Concomitant medications	X	X	X			X					X			X
AE, SAE		X	X	X	X	X	X	X	X	X	X	X	X	X
Download CGM		X				X					X			X
Wash-out period							X	X	X	X				
Phone contact DSSK			X	X	X				X		X	X	X	
Phone contact Dietitian			X	X	X						X	X	X	

10.1 Screening and information

Screening for study participants will we done in the clinics own registers and/or The National Diabetes Register. Advertising in the local press, waiting rooms at the hostpital etc. will also be done.

The subjects will be given a brief overview of the study at a clinical visit and written information approved by ethical committee will also be sent/given to the patient. If an invitation to participate in the study will be sent home by post to the subjects, an informative letter will be added toghether with the patient information that explains why they have received the invitation and inform that they will be contacted by phone within a month.

10.2 Visit 1 – Information and inclusion (week 1-4)

Patients will be permitted to ask questions about the study after reading the written information and receive further explanation if necessary. If the patient gives their written and verbal informed consent to participate, inclusion/exclusion criteria will be assessed.

10.3 Visit 2 – Run-in period (week 1-4)

The following variables will be measured and recorded at this visit:

-Age, sex
-HbA1c
-Weight, Length, BMI
-Blood lipids and apolipoproteins
-Systolic and diastolic blood pressure
-Insulin dose
-Concomitant medications
-Diabetes onset
-Smoking (current, previous, never)
-Concomittant medications
-Previous laser photocoagulation of the retina
-Previous myocardial infarction
-Previous bypass-graft
-Previous PCI
-Amputation
-Previous diabetic foot ulcer
-Current diabetic foot ulcer
-Number of severe hypoglycaemias last year
-Number of severe hypoglycaemias last 5 years
-Dietary inteview (performed by dietitian)
-Physical activity questionnaire
-Hypoglycaemia confidence questionnaire
-Food record 4 days to be filled in by the subject. They will also record insulin doses during this period.
Blinded CGM will be set for all participants to be performed during 2 weeks.

10.4 Visit 3 – Randomization and start of diet 1 (week 5)

Subjects that meet all inclusion and no exclusion criteria and has performed the 2 weeks blinded CGM and completed the food record will be randomized. Subjects will be randomized to receive either a diet with a moderate amount of carbohydrates or a traditional diabetic diet.

The following variables will be measured and recorded:

-HbA1c
-Weight, BMI
-Blood lipids and apolipoproteins
-Systolic and diastolic blood pressure
- DTSQs questionnaire
-Hypoglycaemia confidence questionnaire
-Physical activity level
-Insulin dose
-Concomitant medications

Information with diabetes nurse about how to manage insulin dosage together with the new diet and information with the dietitan about the intervention diet, individualized diet plan etc. A doctor will also be consulted about the insulin doses regarding the new diet. The subjects will also receive information how to make a food record during the 4 days during the last week of the intervention.

CGM-data will be downloaded and the subjects will get instructions of how to get set for the next CGM-measurement period.

10.5 Follow-up (telephone) the day after intervention start, week 5

Information/feedback with diabetes nurse about how to manage insulin dosage together with the new diet.
Information/feedback with dietitan about the intervention diet, individualized diet plan etc.

10.6 Follow-up (telephone) 4 days after intervention start, week 5

Information/feedback with diabetes nurse about how to manage insulin dosage together with the new diet.
Information/feedback with dietitan about the intervention diet, individualized diet plan etc.

10.7 Follow-up, 7 days after intervention start (telephone), week 6

Information/feedback with diabetes nurse about how to manage insulin dosage together with the new diet.
Information/feedback with dietitan about the intervention diet, individualized diet plan etc.

10.8 Follow-up, 14 days after intervention start, week 7

Information/feedback with diabetes nurse about how to manage insulin dosage together with the new diet.
Information/feedback with dietitan about the intervention diet, individualized diet plan etc.

Blinded CGM will be performed during the last 2 weeks of this 4 week diet period and a rέminder and instructions about this with diabetes nurse/dietitian. Diet interview and follow-up with dietitian and remainder of the food record coming up.

10.9 Visit 4 – Intervention 1 finished, week 8

The following variables will be measured and recorded:
-HbA1c
-Weight, BMI
-Blood lipids and apolipoproteins
-Systolic and diastolic blood pressure
- DTSQs questionnaire
-Hypoglycaemia confidence questionnaire

-Physical activity level
-Insulin dose
-Concomitant medications

Collecting and checking the diet record together with the subjects.
CGM-data will be downloaded.

10.10 Wash-out period, telephone contact at week 11

The diabetes nurse or dietitian will call the subject to remind them/instruct them how and when to set the CGM system. Blinded CGM will be performed during the last 2 weeks of this 4 week period (week 11-12).

10.11 Visit 5 – Intervention 2 start, week 13

The following variables will be measured and recorded:
-HbA1c
-Weight, BMI
-Blood lipids and apolipoproteins
-Systolic and diastolic blood pressure
- DTSQs questionnaire
-Hypoglycaemia confidence questionnaire
-Physical activity questionnaire
-Insulin dose
-Concomitant medications

Information with diabetes nurse about how to manage insulin dosage together with the new diet and information with the dietitan about the intervention diet, individualized diet plan etc. A doctor will also be consulted about the insulin doses regarding the new diet.The subjects will also receive information how to make a food record during the 4 days during the last week of the intervention.

CGM-data will be downloaded and the subjects will get instructions of how to get set for the next CGM-measurement period.

10.12 Follow-up (telephone) the day after intervention start, week 13

Information/feedback with diabetes nurse about how to manage insulin dosage together with the new diet.
Information/feedback with dietitan about the intervention diet, individualized diet plan etc.

10.13 Follow-up (telephone) 4 days after intervention start, week 13

Information/feedback with diabetes nurse about how to manage insulin dosage together with the new diet.
Information/feedback with dietitan about the intervention diet, individualized diet plan etc.

10.14 Follow-up, 7 days after intervention start (telephone), week 14

Information/feedback with diabetes nurse about how to manage insulin dosage together with the new diet.
Information/feedback with dietitan about the intervention diet, individualized diet plan etc.

10.15 Follow-up, 14 days after intervention start, week 15

Information/feedback with diabetes nurse about how to manage insulin dosage together with the new diet.
Information/feedback with dietitan about the intervention diet, individualized diet plan etc.

Blinded CGM will be performed during the last 2 weeks of this 4 week diet period and a reminder and instructions about this with diabetes nurse/dietitian. Diet interview and follow-up with dietitian and remainder of the food record coming up.

10.16 Visit 6 – Intervention 2 finished, week 16

The following variables will be measured and recorded:
-HbA1c
-Weight, BMI
-Blood lipids and apolipoproteins
-Systolic and diastolic blood pressure
- DTSQs questionnaire
-Hypoglycaemia confidence questionnaire
-Physical activity level
-Insulin dose
-Concomitant medications

Collecting and checking the diet record together with the subjects.
CGM-data will be downloaded and the gadget and sensor returned by the patient.

11 Asessment of Safety

11.1 Hypoglycaemia, ketons, ketoacidosis and gastrointestinal symptoms

The number of severe hypoglycaemic events, defined as unconsciousness due to hypoglycaemia or need of assistance from another person to resolve hypoglycemia, will be recorded. The time with low glucose values (<3.0 och <3.9) will be measured by CGM and recorded. Ketons, insulindoses and occurance of ketoacidosis will also be recorded. Gastrointestinal AE:s will also be recorded at each visit.

11.2 Adverse Events (AE)

11.2.1 Definition of AE

An AE is defined as any untoward medical occurrence in a clinical investigation subject administered or intended for administration of a pharmaceutical product, including placebo. An AE does not necessarily have a causal relationship with treatment. An AE can be:

- Any unfavorable and unintended sign (including an abnormal laboratory finding), symptom, or disease temporally associated with the use of a medicinal product, whether or not considered related to the medicinal product. This may include changes in laboratory values, diagnostic test results or physical examination (including rectal examination) findings
- Any new disease or exacerbation of an existing disease. Medical conditions/diseases present prior to starting the study are considered AEs if they worsen during the study
- Any deterioration in measurements of laboratory values or other clinical tests (e.g., ECG or x-ray) that results in symptoms, a change in treatment or discontinuation from study drug

- Recurrence of an intermittent medical condition (e.g. headache) not present at baseline

11.2.2 Hypoglycaemias

Hypoglycaemia is an AE by definition, but non-severe hypoglycaemias will not be considered an AE in this study since non-severe hypoglycaemias are common among type 1 diabetic patients in clinical practice. Non-severe hypoglycaemias will not be recorded on the AE pages of the CRF.

11.2.3 Reporting of Adverse Events (AE)

Recording and follow up of AEs will be made from the time of the first study related activity until the completion of the final study visit (SAEs will be followed up until resolved or the event or sequelae stabilizes).
AEs will be recorded on the AE pages of the CRF. For each AE, the following information will be recorded:

- AE (e.g. headache)
- Start/stop date
- Severity
- Action taken
- Relationship to CGM/self-measurement of blood glucose
- Outcome
- Seriousness

A cluster of signs and symptoms that results from a single cause should be reported as a single AE (e.g., fever, elevated WBCs, cough, abnormal chest x-ray, etc. should all be reported as "pneumonia").

11.3 Serious Adverse Events (SAE)

11.3.1 Definition of SAE

An SAE is any medical occurrence at any dose that:

- Results in death
- Is life-threatening (i.e. the subject was at immediate risk of death from the AE as it occurred. This does not include an event that, had it occurred in a more severe form or was allowed to continue, might have caused death)
- Requires inpatient hospitalisation or prolongs existing hospitalisation
- Results in persistent or significant disability/incapacity
- Is a congenital anomaly/birth defect (in the child of a subject who was exposed to the study medication)
- Is a medically important event or reaction (see below)

Other important medical events that may not be immediately life-threatening or result in death or hospitalisation but may, based on appropriate medical judgment, jeopardise the subject or require intervention to prevent one of the outcomes in the definition of SAE listed above should also be considered SAEs. Examples of such events are intensive treatments in an emergency room or at home for allergic bronchospasm, blood dyscrasias or seizures that do not result in hospitalisation, or development of drug dependency or drug abuse. These events may be considered to need rapid reporting by the Sponsor to competent authorities.

11.3.2 Reporting of SAE

All SAEs will be recorded.
All SAEs will be recorded on the AE pages of the CRF. Subjects with SAEs must be followed until the event resolves, or the event or sequelae stabilise.

12 Statistics

12.1 Populations

12.1.1 Full analysis set (ITT-population)

The full analysis set (ITT-population) consists of all randomised subjects who has registered CGM-data for both study periods.

12.1.2 Per-Protocol population

The Per-Protocol population (PP-population) consists of all subjects in the ITT-population who has registered CGM-data for both study periods and has completed the diet record of both study periods and has complied to the diets and has no other protocol deviation that may have had any significant affect on the primary endpoint outcome. The PP-population is defined at the clean-file meeting before the database is locked.

12.1.3 Safety Population

The safety population consists of all randomized subjects who received the diet with moderate amount of carbohydrates during any time period. In the safety analysis a subject will belong to the treatment given not to the randomised treatment.

12.1.4 Sensitivity analysis

Sensitivity analysis will be used for the primary endpoint using multiple imputation.

12.2 General Statistical Methodology

The study design is a randomized cross-over study with a 4 weeks wash-out period.The statistical tests for the cross-over design will be applied.
The analyses of the normally distributed variables will be analysed by using generalized linear models, adjusting for period and patient effects, with sequence, patient(sequence), period and treatment as class variables. Least Square (LS) means with 95% Confidence Intervals (CIs) and associated p-values will be presented from these models.
The analyses of the variables distributed by binomial, Poisson or negative binomial distribution will be analysed in similar way by using generalised estimating equations, using the distribution that fits the data best. The Odds Ratios (OR) with 95% CIs and associated p-values will be presented from these models.
In case the distribution of the effect variables is not appropriately fit with any of the specified distribution above, non-parametric methods for cross-over design will be used. The test between treatment with respect to continuous variables will be tested by Mann-Whitney U-test in that case. The test between two groups with respect to dichotomous variables will be tested by using Fisher's exact test and Mantel-Haenszel Chi-2 test for ordered categorical data.

The missing data will be handled by using multiple imputation method with 50 study samplings on all patients randomized by using demographics, baseline characteristics, baseline comorbidities and glucose values at run-in as imputation variables.

All significance tests will be two-sided and conducted at the 5% significance level. All analyses will performed by using SAS statistical software version 9.4 or later.
The main primary and secondary analyses will be done on the ITT and PP population.

12.3 Efficacy analyses

12.3.1 Primary efficacy analysis

The primary efficacy analysis is the analysis of mean glucose levels at weeks after each diet period between "moderate carbohydrate diet" and "traditional diabetic diet" for ITT population.
The methods described in the general statistical methodology above will be applied. The significance of carry-over effect in the study will be tested by introducing an interaction term between the period and the treatment in the model for the primary variable.
Primary efficacy analysis will also be performed on the PP population.
All tests will be two-sided test and conducted at the 5% significance level.

All measurements obtained after rescue therapy should be excluded in all efficacy analyses.

12.3.2 Secondary efficacy analyses

All the secondary efficacy analyses will performed on all secondary efficacy variables according to the principles given in section 12.2, General Statistical Methodology above.
All secondary efficacy analyses will be two-sided, conducted at the 5% significance level on the ITT- and PP-population.

12.4 Statistical Analysis Plan

A Statistical Analysis Plan that contains a detailed description of all planned analysis will be written and signed before the database is locked.

12.5 Sample Size Calculation

Estimations of expected SD were made with data from our earlier studys with measures of difference in mean glucose level with blinded CGM (ref) and with different scenarios for differences in mean glucose level and different examples of SD.

With Wilcoxon Signed Rank test and a power = 0.80 we need the following number of subjects in the study, two-sided test, alfa 0,05:

1. For a difference of =1 mmol/L and SD=2,5 we need to include 54 subjects
2. For a difference of =1,5 mmol/L and SD=3,5 we need to include 48 subjects
3. For a difference of =2 mmol/L and SD=4,5 we need to include 45 subjects

SD will probably be around 2,5 mmol/L and in that case we will have enough power to study relatively small effects on 1 mmol/L in mean glucose level if 50 subjects is included. The examples above shows that even if SD is higher we will still have the power to detect more significant effects like 2,0 mmol/L difference in mean glucose level if 50 subjects will be included.
The calculations has been performed by Aldina Pivodic, statistcian, Statistiska konsultgruppen, Gothenburg.

12.6 Safety analyses

All safety analyses will be performed on the safety population.
All AE and SAE will be coded using the MedDRA dictionary and tabulated by treatment group.
Number of events, number of patients with events and percentage of patients with events will be given for:

- All events
- All SOC-classes
- All PT-codes within each SOC -code.

13 Premature termination of the trial

The Sponsor or the Investigator may decide to stop the trial or part of the trial at any time.
If a trial is prematurely terminated or suspended, the Investigator should promptly inform the subjects and ensure appropriate therapy and follow-up. Furthermore, the Investigator should promptly inform the IEC (Independent Ethics Committee) and provide a detailed written explanation. The pertinent regulatory authorities should be informed according to national regulations.

14 Data Handling and Record Keeping

14.1 Data Collection

Study data will be collected using electronic Case Record Forms (eCRF). No personal identifiers will be recorded in the eCRF but only the anonymous Suject ID number assigned to each subject in the study will be used. CGM data will be downloaded by the investigator sites using software supplied with the CGM device. No personal identifiers other than the anonymous Subject ID will be recorded in the CGM system. The downloaded data files will be used for study analysis. CGM mean and standard deviation values will also be recorded on the CRF, but the values recorded on the CRF will only be used in the event any electronic files cannot be obtained due to technical issues during the download process.

14.2 Data Management

A Data Management Plan (DMP) will be written to detail data management activities during the trial.

14.2.1 Study Database & Data Entry

An eCRF will be set up for data entry by Investigator site personnel.

14.2.2 Clean File & Database Lock

Once all study data has been collected and entered, the database will be reviewed for completeness, accuracy and consistency. At a formal Clean File meeting the database will be declared locked, after which point the database will be write protected and the analysis start.

14.3 Data Retention & Archiving

Study site should keep study documents and records, including printout copies of the eCRF and CGM records, for 10 years after the study ends.
After the study has been closed, printout copies of the eCRF and raw datasets (eCRF, central lab, and CGM data) will be transferred archived in accordance with data archiving requirements.

15 Access to Source Data/Documents

The investigator will permit trial-related monitoring, audits, and regulatory inspection(s), providing direct access to source data/documents.

16 Ethics

The trial will be conducted in accordance with the Declaration of Helsinki Ethical Principles for Medical Research Involving Human Subjects. The protocol is subject to review and approval by relevant ethics committee. Each subject must be informed that participation in the study is voluntary and that he/she may withdraw from the study at any time and that withdrawal of consent will not affect his/her subsequent medical treatment or relationship with the treating physician.

17 Insurance

Subjects are insured according to the Swedish patient insurance scheme.

18 Compensation

The subjects will recieve a economic compensation for their trouble of participating in the study in the form of giftcards of 300 x 3 (900) SEK. The giftcards will be administered after the study is finished.

19 Publication Policy

The trial will be posted on https://clinicaltrials.gov before trial start.
The results of the trial will be published by the Investigators in an international scientific journal.

20 References

1. DCCT Study Group. The Effect of intensive treatment of diabetes on the development and progression of long-term complications in insulin-dependent diabetes mellitus. The New England Journal of Medicine. 1993;329:977-986.

2. Nathan DM, Cleary PA, Backlund JY, Genuth SM et. al. (Diabetes Control and Complications Trial/Epidemiology of Diabetes Interventions and Complications (DCCT/EDIC) Study Research Group.) Intensive diabetes treatment and cardiovascular disease in patients with type 1 diabetes. The New England Journal of Medicine. 2005;353:2643-2653.

3. Lind M, Svensson AM, Kosiborod M, Gudbjörnsdottir S, Pivodic A, Wedel H, Dahlqvist S, Clements M, Rosengren A. Glycemic control and excess mortality in type 1 diabetes. N Engl J Med. 2014;371(21):1972-82.

4. Ahlén E., Pivodic A., Wedel H., Dahlqvist S., Kosiborod M. Lind M. Glycemic control, renal complications and current smoking in relation to excess risk of mortality in persons with type 1 diabetes. J Diabetes Sci Technol. 2016 Jun 14. [Epub ahead of print]

5. Hanas R. Type 1 diabetes in children, adolescents and young adults — how to become an expert on your diabetes. 4th ed. London: Class Publishing, 2008. 11 of 15

6. Asplund, K., et al., Mat vid diabetes - En systematisk litteraturöversikt. 2010, SBU - Statens beredning för medicinsk utvärdering: Stockholm.

7.Socialstyrelsen. Kost vid diabetes – en vägledning till hälso- och sjukvården. Västerås : Socialstyrelsen, 2011.

8. Nielsen JV et al. Low carbohydrate diet in type 1 diabetes, long-term improvement and adherence: A clinical audit. Diabetology & Metabolic Syndrome. 2012;4(1):23.

9. Krebs JD et al. A randomised trial of the feasibility of a low carbohydrate diet vs standard carbohydrate counting in adults with type 1 diabetes taking body weight into account. Asia Pacific journal of clinical nutrition. 2016;25(1):78-84.

Protocol Amendment

Number: 1

Date: 2017-11-06

The effect of carbohydrate content in the diet on mean glucose levels in type 1 diabetes

Sponsor:

Marcus Lind, MD, PhD, Sahlgrenska Academy, University of Gothenburg, Gothenburg, Sweden and NU-Hospital Organization, Uddevalla, Sweden

Overall changes in the protocol:

The continuous glucose monitoring (CGM) system DexComG4 (DexCom Corporation) will be changed to another similar system also measuring blood glucose in a comparable way named: FreestyleLibre Pro (Abbott). This will be changed in all places that DexComG4 is mentioned in the protocol.

Section(s) of protocol to be amended:

**Previous text**	**Revised text**
1.1. Title page.<br><br>CGM System: DexComG4 (DexCom Corporation)	1.1. Title page.<br><br>CGM System: FreestyleLibrePro (Abbott)
2.3. List of Abbreviations and definitions of terms.<br><br>(no previous text)	2.3. List of Abbreviations and definitions of terms.<br><br>FGM=Flash glucose monitoring
3.4.3.<br><br>DexCom Corporation will provide the DexCom G4 systems (CGM) and sensors during the trial.	3.4.3.<br><br>Abbott will provide the FreestyleLibre Pro systems and sensors during the trial.
4. 7.3. Randomization. DexCom Corporation will provide the DexCom G4 systems (CGM) and sensors.	4.7.3. Randomization.<br><br>Abbott will provide the FreestyleLibre Pro systems and sensors.

Previous text	Revised text
5. 8.1.2 Exclusion criteria.<br><br>7. Continuous use of paracetamol use where not other pain killers can be used (because paracetamol use can affect the accuracy of the Dexcom CGM system).	5.8.1.2 Exclusion criteria.<br><br>Withdraw the text (because we no longer use the DexCom G4).
6. 9.1. Treatment procedures<br><br>All patients will wear a sensor and a blinded CGM system during a run-in period and during 2 weeks of the intervention periods, and during 2 weeks of the wash-out period. In total they will use the CGM system for 8 weeks.	6. 9.1. Treatment procedures<br><br>All patients will wear a sensor and a blinded CGM system during a run-in period of 4 weeks and continue for the whole study period, a total of 16 weeks.
7.10.3 Visit 2 – Run-in period (week 1-4)<br><br>Blinded CGM will be set for all participants to be performed during 2 weeks.	7.10.3 Visit 2 – Run-in period (week 1-4)<br><br>Blinded CGM will be set for all participants to be performed from start of this period and to the end of the study. Subjects will recieve all material (sensors and start kit) and be learned how to change sensors every 14th day.
8. 10.4. Visit 3 –Randomization and start of diet 1 (week 5)<br><br>(none)	8.10.4. Visit 3 –Randomization and start of diet 1 (week 5)<br><br>-Ketone levels (measured twice a week)
9.10.8 Follow-up, 14 days after intervention start, week 7<br><br>Blinded CGM will be performed during the last 2 weeks of this 4 week diet period and a reminder and instructions about this with diabetes nurse/dietitian. Diet interview and follow-up with dietitian and remainder of the food record coming up.	9.10.8 Follow-up, 14 days after intervention start, week 7<br><br>Diet interview and follow-up with dietitian and remainder of the food record coming up.
10. 10.9. Visit 4 – Intervention 1 finished, week 8<br><br>(none)	10.10.9.Visit 4 – Intervention 1 finished, week 8<br><br>Collect ketone diaries

Previous text	Revised text
11.10.10 Wash-out period, telephone contact at week 11<br><br>The diabetes nurse or dietitian will call the subject to remind them/instruct them how and when to set the CGM system. Blinded CGM will be performed during the last 2 weeks of this 4 week period (week 11-12).	11.10.10 Wash-out period, telephone contact at week 11<br><br>The diabetes nurse or dietitian will call the subject to remind/instruct them how and when to change the CGM sensor.
12. 10.11. Visit 5 – Intervention 2 start, week 13<br><br>(none)	12. 10.11. Visit 5 – Intervention 2 start, week 13<br><br>-Ketone levels (measured twice a week)
13.10.15. Follow-up, 14 days after intervention start, week 15<br><br>Blinded CGM will be performed during the last 2 weeks of this 4 week diet period and a reminder and instructions about this with diabetes nurse/dietitian. Diet interview and follow-up with dietitian and remainder of the food record coming up.	13.10.15. Follow-up, 14 days after intervention start, week 15<br><br>Diet interview and follow-up with dietitian and remainder of the food record coming up and change of CGM-sensor.
14.10.16. Visit 6 – Intervention 2 finished, week 16<br><br>(none)	14.10.16. Visit 6 – Intervention 2 finished, week 16<br><br>Collect ketone diaries
15.12.2 General Statistical Methodology.<br>(none)	15.12.2 General Statistical Methodology.<br><br>If data from the blinded CGM-sensor is missing (because of technical problems or participants forgetting to put on a new sensor) and the participant has a regular, non-blinded CGM/FGM-device with data from the study period, we will use this data in the analyses and carefully document it.

Reasons for Amendments:

1. We will use the CGM-system FreestyleLibrePro instead of the CGM-system DexCom G4 system. This is a similar system and it is also more user friendly for the patients and does not require calibration every morning and evening and the sensors only need to be changed every 14th day. Therefore we will let the participants wear the CGM sensors during the whole study period (16 weeks) instead of only the last 14 days of run-in, diet period 1 and 2 and wash-out (8 weeks). We will use the data to explore the mechanisms of the diets, but we keep the main endpoints (comparing the last 14 days of the diet periods when they have adapted to their new diets).

2. If the participants in the study already have a CGM or flash glucose monitoring (FGM)-system and if data is missing from the blinded CGM-system (because of technical problems or if they had forgot to put on a new sensor) we will download and use the data from the participants own sensor in the analyses. This will be recorded.

3. We do not find it necessary for the participants to measure blood ketones two times a day as in the first version of the protocol. This is because the risk of ketoacidosis in this study is considered to be very small. The measurements of ketones is mainly maid to understand if these values changes of the different diets, rather than avoiding ketoacidosis. So we have changed the amount of measurements to twice a week (Mondays and Thursdays) and if they have symptoms of ketoacidosis.

Signed agreement to the Amendment:

I agree to the terms of this Protocol Amendment.

6/11 - 2017 Marcus Lind

Date
(day month year)

Principal investigator
Marcus Lind

**Protocol Amendment**	
Number:	2
Date:	2018-03-15

The effect of carbohydrate content in the diet on mean glucose levels in type 1 diabetes

Sponsor:

Marcus Lind, MD, PhD, Sahlgrenska Academy, University of Gothenburg, Gothenburg, Sweden and NU-Hospital Organization, Uddevalla, Sweden

Section(s) of protocol to be amended:

1. 1.1 Treatment procedures

Previous text:

All subjects in the trial will be instructed regarding basic information on insulin dosing, such as bolus correction, types of food elevating glucose levels and the effect of physical activity on glucose control. This information will be provided at the same level as in clinical practice for patients with type 1 diabetes, i.e. to guarantee that all subjects have basic skills for dosing insulin before the interventions begins.

All patients will wear a sensor and a blinded CGM system during a run-in period of 4 weeks and continue for the whole study period, a total of 16 weeks. Care-givers will down-load data from the CGM system at all clinical visits. At clinical visits and at telephone contacts the research staff will discuss the current diet and try to motivate the patient to keep the diet in accordance to the protocol. Further, the research staff will perform a general check that the patient takes a reasonable amount of basal insulin, both from a safety perspective and efficacy perspective. In accordance the research staff will check if the patient performs adequate adjustments of prandial insulin doses and adjust these if needed.

Regarding the subjects that are eating the diet low in carbohydrates the insulin regimen needs to be adjusted when the subjects start with the diet. They will probably need to lower their insulin doses by about 20-30 % compared to when they are eating normal amounts of carbohydrates in the diet, but this will be calculated individually depending on their regular diet as well as other affecting factors. And it is also possible that the lowering of insulin doses is just initial, and that they will need to use higher doses again after the first days of the new diet.

hypoglycaemia or any fasting glucose level below 4.0 mmol/l the basal insulin dose shall be increased by 2 units in the FPG interval 6.5-10 mmol/l and 4 units if the mean level has been above 10 mmol/l. If the FPG level repeatedly has been 3-4 mmol/l or nocturnal hypoglycaemia has appeared or at any time FPG <3.0 mmol/l the basal insal insulin dose shall be reduced by 2-4 units. For insulin degludec the same algorithm shall be used every 5th day. If the patient or care-giver wants to reduce an insulin dose even earlier than every 3rd day due to safety reasons such as fear of hypoglycaemia this shall be made, recommended by 2-4 units. If the patient repeatedly have had clear postprandial hyperglycaemia after the evening meal and high glucose levels at bed time and the evening meal prandial dose is decided to be increased, this can be another reason for awaiting increasing the insulin dose although FPG-levels are elevated. If the patient has daytime hypoglycaemias that are clearly judged to be due to too high basal insulin dose that not can be compensated by e.g. suitable meals an individual judgement must be made by the patient/care-giver if the basal insulin dose shall be reduced/not increased although FPG may be increased. In summary the above basal insulin algorithm is a recommendation to follow, but safety must always be prioritised and if the patient/care-giver judges that the algorithm should not be followed from a safety perspective in certain instances this should be done.

For persons on insulin pumps a similar algorithm shall be used every 3rd day. The basal insulin dose overnight shall be increased by an average by 0.1 units/hour if FPG 6.5-10 mmol/l and by an average of 0.2 units/hour if FPG >10 mmol/l. In correspondence the basal overnight insulin dose shall be reduced by 0.1-0.2 units/hour at nocturnal hypoglycaemia or low FPG-levels (see description above for basal insulins). Also in correspondence the patient and care-giver always have the right from a safety perspective to reduce or skip enhancing the basal insulin dose if fear of hypoglycaemia. For patients on insulin pumps individual judgement will be made if the basal insulin dose daytime shall be adjusted. During weeks 2-4 in each treatment phase the above insulin adjustments will be made every 5th day by the patient him/herself. The same general recommendations for deviating from this algorithm are the same as those described above for basal insulin injections.

1.1.3 Meal time insulin dose adjustments

The patient will continue with the same strategy for dosing meal time insulin as used earlier in clinical practice. If the patient e.g. has used carbohydrate counting by weighing the food this will be continued. If carbohydrate counting has been used by visually estimating carbohydrates this will be continued. If the patient has performed an overall estimation of meal time insulin dose from earlier experience for a certain type of food this will be used. The patient will with the used method try to dose meal time insulin in accordance with the current meal in the study. The nutritionist will inform what type of food contains carbohydrates and give a similar support for both types of diets. The physician/diabetic nurse will at planned telephone contacts give support from glucose curves whether a certain meal time doses seem to have been under- or overdosed to take into consideration for meal time insulin dosing. The advices will be at the same level in both treatment phases. The patient and care giver should stribe for obtaining a glucose level that is <10 mmol/l 2 hours after meals and <7 mmol/l before meals. If the glucose levels are repeatedly over these levels it indicates that a higher meal-time insulin dose is needed for that specific meal.

2.

Previous text:

10.3 Visit 2 – Run-in period (week 1-4)

-Blood lipids and apolipoproteins

Revised text:

10.3 Visit 2 – Run-in period (week 1-4)

Removed the text. (This has also been removed from table 10. Trial procedures).

Reasons for Amendments:

1. To make the protocol more clear, and to make it easier for both subjects and study personnel, and to be able to make the insulin adjustment part in the study as similar as possible between the two diet interventions we have made some adjustments in the text regarding insulin adjustments and blood glucose measurements.

   We also made a correction in this section about weeks with CGM which is 14 and not 16.

   An amendment about time frame for clinical visits and phone contacts is also added.

2. When reading the protocol through again we reckon that this was wrong, blood lipids and apolipoproteins should only be measured before and after each diet intervention.

Signed agreement to the Amendment:

I agree to the terms of this Protocol Amendment.

15/3-2018

Date
(day month year)

Principal investigator
Marcus Lind

**Protocol Amendment**	
Number:	3
Date:	2018-08-15

The effect of carbohydrate content in the diet on mean glucose levels in type 1 diabetes

Sponsor:

Marcus Lind, MD, PhD, Sahlgrenska Academy, University of Gothenburg, Gothenburg, Sweden and NU-Hospital Organization, Uddevalla, Sweden

Section(s) of protocol to be amended:

Previous text:

4.6 Investigator and site

Marcus Lind, MD, PhD, NU-hospital Organization, Uddevalla, Sweden. Uddevalla Hospital will be the place were the study will be conducted.

Revised text:

4.6 Investigators and sites

Marcus Lind, MD, PhD, NU-hospital Organization, Uddevalla, Sweden. Uddevalla Hospital.

Staffan Hederoth, Ersta hospital, Stockholm

Ulf Rosenqvist, Motala hospital, Motala

5. INTRODUCTION & BACKGROUND INFORMATION

5.1 Background

Previous text:

The current trial is of cross-over design and 12 weeks in duration, where patients will be randomized to; 1) a diet with moderate carbohydrate content for 4 weeks and 2) traditional diabetic diet with low GI for 4 weeks and a wash-out period for 4 weeks. The primary endpoint is the effect on mean glucose levels measured by CGM.

Revised text:

6. INTRODUCTION & BACKGROUND INFORMATION

6.1Background

The current trial is of cross-over, multicenter design and 12 weeks in duration, where patients will be randomized to; 1) a diet with moderate carbohydrate content for 4 weeks and 2) traditional diabetic diet with low GI for 4 weeks and a wash-out period for 4 weeks. The primary endpoint is the effect on mean glucose levels measured by CGM.

Previous text:

7. TRIAL DESIGN

7.1Design

A randomized, non-blinded, cross-over clinical trial.

Revised text:

7. TRIAL DESIGN

7.1Design

A randomized, non-blinded, cross-over clinical multicenter trial.

Reasons for Amendments:

1. We have added two sites to the study protocol.
2. We have added that it is a multicenter study

Signed agreement to the Amendment:

I agree to the terms of this Protocol Amendment.

15 Aug 2018

Date
(day month year)

Principal investigator
Marcus Lind

Protocol Amendment
Number: 4
Date 2019/09/09

The effect of carbohydrate content in the diet on mean glucose levels in type 1 diabetes

Sponsor:

Marcus Lind, MD, PhD, Sahlgrenska Academy, University of Gothenburg, Gothenburg, Sweden and NU-Hospital Organization, Uddevalla, Sweden

Section(s) of protocol to be amended:

Previous text	Revised text
**4.6 Investigatora and site**<br><br>Marcus Lind, MD, PhD, NU-hospital Organization, Uddevalla, Sweden. Uddevalla Hospital.<br><br>Staffan Hederoth, Ersta hospital, Stockholm<br><br>Ulf Rosenqvist, Motala hospital, Motala	**4.6 Investigatora and site**<br><br>Marcus Lind, MD, PhD, NU-hospital Organization, Uddevalla, Sweden. Uddevalla Hospital.<br><br>Staffan Hederoth, Ersta hospital, Stockholm<br><br>Ulf Rosenqvist, Motala hospital, Motala<br><br>Magnus Löndahl, Lund Hosptial, Lund

Reason for Amendment:

One Site added to the study protocol

Signed agreement to the Amendment:
I agree to the terms of this Protocol Amendment.

9/9-2019 Marcus Lind

Date
(day month year)

Principal investigator
Marcus Lind

Protocol Amendment
Number: 5
Date 2021/11/29

The effect of carbohydrate content in the diet on mean glucose levels in type 1 diabetes

Sponsor:

Marcus Lind, MD, PhD, Sahlgrenska Academy, University of Gothenburg, Gothenburg, Sweden and NU-Hospital Organization, Uddevalla, Sweden

Section(s) of protocol to be amended:

Previous text	Revised text
**4.6 Investigators and site**<br><br>Marcus Lind, MD, PhD, NU-hospital Organization, Uddevalla, Sweden. Uddevalla Hospital.<br><br>Staffan Hederoth, Ersta hospital, Stockholm<br><br>Ulf Rosenqvist, Motala hospital, Motala<br><br>Magnus Löndahl, Lund Hospital, Lund	**4.6 Investigators and site**<br><br>Marcus Lind, MD, PhD, NU-hospital Organization, Uddevalla, Sweden. Uddevalla Hospital.<br><br>Staffan Hederoth, Ersta hospital, Stockholm<br><br>Ulf Rosenqvist, Motala hospital, Motala<br><br>Magnus Löndahl, Lund Hospital, Lund<br><br>Sara Hallström, Östra hospital, Gothenburg

Reason for Amendment:

One Site added to the study protocol

Signed agreement to the Amendment:
I agree to the terms of this Protocol Amendment.

29/11-2021 Marcus Lind

Date
(day month year)

Principal investigator
Marcus Lind
