# Supplementary material for: The effect of carbohydrate intake on glycaemic control in individuals with type 1 diabetes: a randomised, open-label, crossover trial
Source: Lancet Reg Health Eur. 2023 Dec 19;37:100799. doi: 10.1016/j.lanepe.2023.100799 (PMC10866914; doi:10.1016/j.lanepe.2023.100799)
Supplement: Supplementary Material 2 [file mmc2.pdf]

Statistical Analysis Plan

FINAL

The effect of carbohydrate content in the diet on the mean blood glucose level in persons with type 1 diabetes

2023-03-22

Authors

Name/Title:	
Mattias Molin / Biostatistician, Statistiska Konsultgruppen Sweden AB	
Signature:	Date
Name/Title: Henrik Imberg / Biostatistician, Statistiska Konsultgruppen Sweden AB	
Signature: Henrik Imberg	Date: 2023-03-22

Approvals

Name/Title:	
Marcus Lind / Professor of Diabetology, University of Gothenburg, senior consultant of diabetes NU-Hospital Group and Sahlgrenska University Hospital/Östra	
Signature:	Date
Name/Title: Sofia Sterner Isaksson / University of Gothenburg and Diabetes NU Hospital Group, Gothenburg	
Signature: Sofia Sterner	Date: 2023.03.22

Statistical Analysis Plan

FINAL

The effect of carbohydrate content in the diet on the mean blood glucose level in persons with type 1 diabetes

2023-03-22

Authors

Name/Title:<br>Mattias Molin / Biostatistician, Statistiska Konsultgruppen Sweden AB<br><br>Signature: .... Date: ....
Name/Title:<br>Henrik Imberg / Biostatistician, Statistiska Konsultgruppen Sweden AB<br><br>Signature: .... Date: ....

Approvals

Name/Title:<br>Marcus Lind / Professor of Diabetology, University of Gothenburg, senior consultant of diabetes NU-Hospital Group and Sahlgrenska University Hospital/Östra<br><br>Signature: .... Date: 22/3 2023
Name/Title:<br>Sofia Sterner Isaksson / University of Gothenburg and Diabetes NU Hospital Group, Gothenburg<br><br>Signature: .... Date: ....

Statistical Analysis Plan

FINAL

The effect of carbohydrate content in the diet on the mean blood glucose level in persons with type 1 diabetes

2023-03-22

Authors

Name/Title:	
Mattias Molin / Biostatistician, Statistiska Konsultgruppen Sweden AB	20230322
Signature:	Date
Name/Title: Henrik Imberg / Biostatistician, Statistiska Konsultgruppen Sweden AB	
Signature:	Date

Approvals

Name/Title:	
Marcus Lind / Professor of Diabetology, University of Gothenburg, senior consultant of diabetes NU-Hospital Group and Sahlgrenska University Hospital/Östra	
Signature:	Date
Name/Title: Sofia Sterner Isaksson / University of Gothenburg and Diabetes NU Hospital Group, Gothenburg	
Signature:	Date

4.7.2 Secondary Efficacy Analyses 15
4.7.3 Exploratory Efficacy Analyses 15
4.8 Safety Analyses 16
4.8.1 Exposure 16
4.8.2 Adverse Events 16
5 Interim Analyses 16
6 Changes of Analysis from Protocol 16
7 Listing of Table, Figures and Listings 17
7.1 Listing of Tables 17
7.2 Listing of Figures 17
7.3 Listing of Listings 18
8 References 18
9 Appendix 18

LIST OF ABBREVIATIONS

Abbreviation	Definition
AE	Adverse event
ATC	The Anatomical Therapeutic Chemical Classification System
CGM	Continuous glucose monitoring
CI	Confidence interval
DTSQc	Diabetes treatment satisfaction questionnaire, change version
DTSQs	Diabetes treatment satisfaction questionnaire, status version
eCRF	Electronic case report form
FAS	Full analysis set
HbA1c	Haemoglobin A1c
HDL	High-density lipoprotein
ICD-10	The 10th revision of the International Statistical Classification of Diseases and Related Health Problems
LDL	Low-density lipoprotein
MAGE	Mean amplitude of glycaemic excursions
MC	Moderate carbohydrate (diet)
PP	Per-protocol
SAE	Serious adverse event
SD	Standard deviation
TD	Traditional diabetes (diet)

1 STUDY DETAILS

1.1 Study Objectives

The primary objective is to examine whether a moderate carbohydrate (MC) diet is more effective than a traditional diabetic (TD) diet with a higher amount of carbohydrates on reducing the mean blood glucose level in patients with type 1 diabetes.

The secondary objectives are to examine whether the moderate carbohydrate diet has a different effect than a traditional diabetic diet on the standard deviation of blood glucose levels, MAGE, time in hypoglycaemia, time in hyperglycaemia, time in range, hypoglycaemia confidence, treatment satisfaction, HbA1c, blood lipids, weight, total insulin dose and ketones in patients with type 1 diabetes.

1.2 Study Design

A randomised, non-blinded, cross-over clinical multicentre trial.

Trial procedure during the run-in phase and trial is schematically shown below:

Week	1–4		5	6	7	8	9	10	11	12	13	14	15	16
Visit	Run-in phase													
Visits at site	1	2	3			4					5			6
Screening/ Information	X													
Informed consent	X													
Randomisation			X											
Blinded CGM		X	X	X	X	X	X	X	X	X	X	X	X	X
Demographics, medical history		X												
HbA1c		X	X			X					X			X
Blood lipids, apolipoproteins			X			X					X			X
Blood pressure		X	X			X					X			X
BMI		X	X			X					X			X

Diet and insulin record		X				X								X
DTSQs, Hypoglycaemia confidence			X			X					X			X
Physical activity		X	X			X					X			X
DTSQc														X
Insulin dose		X	X			X					X			X
AE, SAE		X	X	X	X	X	X	X	X	X	X	X	X	X
Download CGM			X			X					X			X
Wash-out period							X	X	X	X				
Phone contact DSSK			X	X	X				X		X	X	X	
Phone contact Dietitian			X	X	X						X	X	X	

All patients used a masked Continuous Glucose Monitoring (CGM) system (Freestyle Libre Pro, ABBOTT) during a run-in period of 2-4 weeks and then continuous for the whole study period, a total of 14-16 weeks. Caregivers collected data from the CGM system at all clinical visits. If patients used CGM in their usual diabetes care they continued to use them, and data were collected from their own CGM device as well.

1.3 Treatment Groups

The diet interventions are a moderate carbohydrate (MC) diet (30 E %) compared to a traditional diabetes (TD) diet with a higher amount of carbohydrates (50 E %).

1.4 Sample Size

The target sample size was set to 52 subjects to detect a clinically relevant difference in mean glucose levels of 1 mmol/L between treatments, assuming a within subjects standard deviation of 2.5 mmol/L, paired T-test, 80% power, significance level α=0.05. The standard deviation was estimated on data from the GOLD cross-over trial (1).

2 STUDY POPULATIONS

2.1 Definition of Study Populations

2.1.1 Full Analysis Set

The Full Analysis Set (FAS) consists of all randomised subjects who has registered CGM-data* for at least one of the study periods.

*The CGM-period is defined as the last 14 calendar days with CGM sensor use in the diet period, excluding measurements obtained within two weeks from the start of the diet period. CGM data by the masked CGM device (Libre PRO) will primarily be used if existing in both treatment phases. If the CGM-data of the blinded device is relatively short (<7 days) and CGM-data from the subjects own device exists in both treatment phases with significantly better coverage this will be used instead. If <3 days of CGM-data exists in both treatment phases with one and the same device the mean and SD from the eCRF will be used if existing in both treatment phases. Finally, if any CGM-data exists in both treatment phases with one and the same device it will be used if >1 day of CGM data exist.

2.1.2 Per Protocol Population

There are 4 different per protocol populations that will be analysed (PP1, PP2, PP3, PP4).

PP1 consists of all subjects in the FAS who have no protocol deviations indicating that they have not received diet advise or complied with the diets and who have measurements of CGM in both treatment phases (including eCRF data) within a time period not significantly deviating from the planned time period.

PP2 consists of all subjects in PP1 who in addition have diet records in both treatment phases confirming that the total amount of carbohydrates deviated between the treatment phases with a lower amount during the treatment sequence aimed at reducing the amount of carbohydrates.

PP3 consists of all subjects in PP2 that in addition have a lower % of the total energy intake constituted of carbohydrates during the treatment sequence with moderately reduced carbohydrate intake.

PP4, the CGM-PP population consists of all subjects in the PP1-population with at least 9 days with CGM data for least 70% of the time, in both diet periods.

The PP-populations are defined at the clean-file meeting before the database is locked.

2.1.3 Safety Population

The safety population consists of all randomised subjects who received the diet with moderate amount of carbohydrates during any time period. In the safety analysis a subject will belong to the treatment given not to the randomised treatment.

3 STUDY VARIABLES

3.1 Baseline Variables

3.1.1 Demographics and Baseline Characteristics

- Age (years)
- Sex
- Diabetes duration (years)
- Smoking (current/previous/never)
- Haemoglobin A1c (HbA1c) (mmol/mol and %)
- Weight (kg)
- Body mass index (BMI) (kg/m^2)
- Systolic blood pressure (mmHg)
- Diastolic blood pressure (mmHg)
- Total cholesterol (mmol/L).
- Low-density lipoprotein (mmol/L)
- High-density lipoprotein (mmol/L)
- Triglycerides (mmol/L)
- Total daily insulin dose (IU)
- Daily mealtime (bolus) insulin (IU)
- Daily basal insulin (U)
- Total daily insulin dose to body weight ratio (IU/kg)
- Physical activity level, first item (1–10)
- Change in physical activity level, second item (yes/no)
- DTSQc (total score)
- DTSQs (total score)
- Hypoglycaemia confidence scale (total score)

3.1.2 Medical and Surgical History

- Previous laser photocoagulation of the retina
- Previous myocardial infarction
- Previous bypass-graft
- Previous percutaneous coronary intervention (PCI)
- Amputation
- Previous diabetic foot ulcer
- Current diabetic foot ulcer
- Number of severe hypoglycaemia events last year
- Number of severe hypoglycaemia events last 5 years

3.1.3 Prior and Concomitant Medications

- Concomitant medications coded according to ATC

3.2 Efficacy Variables

3.2.1 Primary Endpoint

The primary endpoint is the difference between the MC and TD diets with respect to the mean blood glucose level measured with CGM during the last two weeks of each diet period. The CGM-period is defined as the last 14 calendar days with CGM sensor use in the diet period, excluding measurements obtained within two weeks from the start of the diet period.

*The CGM-period is defined as the last 14 calendar days with CGM sensor use in the diet period, excluding measurements obtained within two weeks from the start of the diet period. CGM data by the masked CGM device (Libre PRO) will primarily be used if existing in both treatment phases. If the CGM-data of the blinded device is relatively short (<7 days) and CGM-data from the subjects own device exists in both treatment phases with significantly better coverage this will be used instead. If <3 days of CGM-data exists in both treatment phases with one and the same device the mean and SD from the eCRF will be used if existing in both treatment phases. Finally, if any CGM-data exists in both treatment phases with one and the same device it will be used if >1 day of CGM data exist.

3.2.2 Secondary Endpoints

Secondary endpoints are the difference between the MC and TD diets with respect to the following variables:

- Standard deviation of blood glucose levels (mmol/L), measured with CGM during the last two weeks of each diet period.*†
- Time above range (%), defined as the proportion of time with blood glucose levels >10.0 and >13.9 mmol/L, respectively. Measured with CGM during the last two weeks of each diet period.*
- Time in range (%), defined as the proportion of time with blood glucose levels 5.5–10.0 and 3.9–10.0 mmol/L, respectively. Measured with CGM during the last two weeks of each diet period.*
- Weight (kg) at the end of each diet period.
- Total cholesterol (mmol/L) at the end of each diet period.
- Low-density lipoprotein (LDL) (mmol/L) at the end of each diet period.
- High-density lipoprotein (HDL) (mmol/L) at the end of each diet period.
- Triglycerides (mmol/L) at the end of each diet period.
- Total daily insulin dose (IU) during the last 14 days of the diet period, calculated as the sum of all insulin doses during the last 14 days of the diet period divided by the number of days for which insulin records were made.
- DTSQc (total scale) at the end of the study (week 16).
- DTSQs (total scale) at the end each diet period.
- Hypoglycaemia confidence scale (total score) at the end of each diet period.

*The CGM-period is defined as the last 14 calendar days with CGM sensor use in the diet period, excluding measurements obtained within two weeks from the start of the diet period. CGM data by the masked CGM device (Libre PRO) will primarily be used if existing in both treatment phases. If the CGM-data of the blinded device is relatively short (<7 days) and CGM-data from the subjects own device exists in both treatment phases with significantly better coverage this will be used instead. If <3 days of CGM-data exists in both treatment phases with one and the same device the mean and SD from the eCRF will be used if existing in both treatment phases. Finally, if any CGM-data exists in both treatment phases with one and the same device it will be used if >1 day of CGM data exist.

3.2.3 *Exploratory Endpoints*

Exploratory endpoints are the difference between the MC and TD diets with respect to the following variables:

- Time in tight range (%), defined as the proportion of time with blood glucose levels 3.9–7.8 mmol/L, measured with CGM during the last two weeks of each diet period. *
- Time in target (%), defined as the proportion of time with blood glucose levels 3.5-7.8 mmol/L, measured with CGM during the last two weeks of each diet period. *
- MAGE (mmol/L), measured by CGM during the last two weeks of each diet period. *
- Coefficient of variation (CV), measured by CGM during the last two weeks of each diet period. *
- HbA1c (mmol/mol) at the end of each diet period.
- Systolic blood pressure (mmHg) at the end of each diet period.
- Diastolic blood pressure (mmHg) at the end of each diet period.
- Apolipoprotein A (g/L) at the end of each diet period.
- Apolipoprotein B (g/L) at the end of each diet period.
- Apolipoprotein B/Apolipoprotein B ratio at the end of each diet period.
- Daily mealtime (bolus) insulin (U) during the last 14 days of the diet period, calculated as the sum of all meal time insulin doses during the last 14 days of the diet period divided by the number of days for which insulin records were made.
- Daily basal insulin (IU) during the last 14 days of the diet period, calculated as the sum of all basal insulin doses during the last 14 days of the diet period divided by the number of days for which insulin records were made.
- Total daily insulin dose to body weight ratio (IU/kg), with the total daily insulin dose calculated as above divided by the body weight at the end of the study period.

*The CGM-period is defined as the last 14 calendar days with CGM sensor use in the diet period, excluding measurements obtained within two weeks from the start of the diet period. CGM data by the masked CGM device (Libre PRO) will primarily be used if existing in both treatment phases. If the CGM-data of the blinded device is relatively short (<7 days) and CGM-data from the subjects own device exists in both treatment phases with significantly better coverage this will be used instead. If <3 days of CGM-data exists in both treatment phases with one and the same device the mean and SD from the eCRF will be used if existing in both treatment phases. Finally, if any CGM-data exists in both treatment phases with one and the same device it will be used if >1 day of CGM data exist.

Exploratory analyses will also be performed regarding CGM-metrics during days of diet registrations as well as correlation analyses between differences in carbohydrate amount and differences in CGM-metrics and other outcome variables between the two treatment phases (see section of exploratory analyses).

Exploratory analyses of the endpoints above under primary, secondary and exploratory endpoints will also be performed per time period during day (06:00-21:59) and night (22:00-05:59).

3.3 Safety Endpoints

3.3.1 Safety Endpoints

Safety endpoints are the difference between the treatments with respect to the following variables:

- Time below range (%), defined as the proportion of time with blood glucose levels <3.0 mmol/l and <3.9 mmol/l, respectively. Measured with CGM during the last two weeks of each diet period.*
- Number of severe hypoglycaemia events, defined as unconsciousness due to hypoglycaemia or need of assistance from another person to resolve the hypoglycaemia, during each diet period.
- Ketone levels (mmol/L, and categorised into ≥0.6 or ≥1.5 mmol/L) during each diet period. Measured four times per week (morning and evening two days per week).
- Number of ketoacidosis events during each diet period.

*The CGM-period is defined as the last 14 calendar days with CGM sensor use in the diet period, excluding measurements obtained within two weeks from the start of the diet period. CGM data by the masked CGM device (Libre PRO) will primarily be used if existing in both treatment phases. If the CGM-data of the blinded device is relatively short (<7 days) and CGM-data from the subjects own device exists in both treatment phases with significantly better coverage this will be used instead. If <3 days of CGM-data exists in both treatment phases with one and the same device the mean and SD from the eCRF will be used if existing in both treatment phases. Finally, if any CGM-data exists in both treatment phases with one and the same device it will be used if >1 day of CGM data exist.

3.3.2 Exposure

Exposure will be measured by the amount of carbohydrates (E %) in the diet according to food diaries. Exposure will also be described and analysed as the total amount (gram) of carbohydrates.

3.3.3 Adverse Events

AEs from the time of the first study related activity until the completion of the final study visit will be collected in the study.

- AE term (according to ICD-10)
- Severe AE
- Related AE
- Serious AE (SAE)

4 STATISTICAL METHODOLOGY

4.1 General Methodology

Descriptive data will be presented using mean and standard deviation, median, minimum and maximum value for continuous variables. Number and percent will be presented for categorical variables.

Statistical methods for crossover trials will be applied using linear, log-linear, or generalised linear mixed effects models. Treatment (diet), period, and randomisation sequence will be included as fixed effects, and subject and random effect. An appropriate model will be selected depending on the type and distribution of outcome as follows:

- Normally distributed variables (CGM mean, CGM SD, weight, MAGE, HbA1c, systolic and diastolic blood pressure) will be analysed using linear mixed effects models.
- Log-normally distributed variables (total cholesterol, LDL and HDL cholesterol, triglycerides, apolipoproteins, and ketone levels) will be analysed using linear mixed effects models on the log-transformed variable. The regression coefficient of the treatment variable be exponentiated to obtain an estimate of the fold-change between treatments. Analysis of ketones will additionally account for left-censoring of ketone levels <0.1 mmol/L.
- Other non-normally distributed numeric variables (time above range, time in range, time below range, insulin dose, DTSQc, DTSQc, and hypoglycaemia confidence scale) will be analysed using linear mixed effects models with robust standard errors (HC3 method) to obtain inferences that are robust against distributional assumptions.
- Binary and count variables (number of severe hypoglycaemia events, ketone levels ≥0.6 mmol/L or ≥1.5 mmol/L, number of ketoacidosis events) will be analysed using generalised linear mixed effects models with Poisson distribution and log-link. Robust standard errors (HC3 method) will be used to obtain inferences that are robust against distributional assumptions. The regression coefficient of the treatment

variable be exponentiated to obtain an estimate of the relative risk of an event between the treatments. In case of non-convergence, e.g., due to too few events, Prescott's test will be used for binary variables with a single measurement per diet period, and linear mixed models with robust standard errors (HC3 method) otherwise.

The Kenward-Roger method for variance estimation and degrees-of-freedom approximation will be used whenever applicable. Carry-over effects will be evaluated through the significance of treatment with period interactions, and by investigation of baseline, run-in and wash-out values.

All CGM-endpoints will be evaluated on the FAS and all PP populations (PP1-PP4). All other endpoints will be evaluated on the FAS and PP-populations (PP1-PP4). All statistical tests will be two-sided and conducted at the 5% significance level. All estimates will be presented with corresponding 95% confidence intervals. To account for multiple testing, a sequential testing procedure will be employed. In case of a significant test for the primary endpoint, the entire probability mass $\alpha=0.05$ will be transferred to the secondary endpoints in the order listed. The test procedure will continue until the first encounter of a non-significant test. All these significant tests will be considered confirmatory.

Statistical analyses will be performed using SAS/STAT Software version 9.4 (SAS Institute, Cary, N.C.).

4.2 Patient Disposition and Data Sets Analysed

The number of subjects included in each of the FAS, PP populations (PP1-PP4), and safety populations will be summarised. The number and percentage of subjects randomised and treated will be presented. Subjects who completed the study and subjects who withdrew from study prematurely will also be presented with a breakdown of the reasons for withdrawal for the FAS, PP populations (PP1-PP4), and safety populations.

4.3 Protocol Violations/Deviations

Major protocol deviations are those that are considered to have an effect on the analysis. A list of potential major protocol deviations will be generated programmatically from the data captured before the clean file meeting. The clinical monitors of the study will review the list and the finalisation of the major protocol deviations will be done at the clean file meeting.

The number of patients with major protocol deviations will be summarised per treatment group.

4.4 Demographics and Baseline Characteristics

Demographics and baseline characteristics will be summarised for the FAS and PP populations (PP1-PP4) and analysed according to the methods described in section "General Methodology" above.

4.5 Medical History

Medical history will be summarised according to ICD-10 for the full analysis set.

4.6 Prior and Concomitant Medications

Prior and concomitant medication will be summarised by higher level anatomical therapeutic classification (ATC) group and generic term for the full analysis set.

4.7 Efficacy Analyses

4.7.1 Primary Efficacy Analysis

The primary efficacy analysis is the comparison of mean blood glucose levels between the moderate carbohydrate diet and traditional diabetes diet on the full analysis set. The mean difference between the diets will be estimated using linear mixed effects models with treatment (diet), period, and randomisation sequence as fixed effects, and subject and random effect. The primary efficacy analysis will also be performed on the PP populations.

4.7.2 Secondary Efficacy Analyses

Secondary efficacy analyses will be performed on the secondary endpoints according to the principles given in section General Methodology above. All secondary efficacy analyses will be two-sided and conducted at the 5% significance level. All CGM-endpoints will be evaluated on the FAS and PP (PP1-PP4) populations. All other endpoints will be evaluated on the FAS and PP-populations. All patients that changed their lipid lowering medication during the study will be excluded from the corresponding efficacy analyses.

4.7.3 Exploratory Efficacy Analyses

Exploratory efficacy analyses will be performed on the exploratory endpoints according to the principles given in section General Methodology above. All exploratory efficacy analyses will be two-sided and conducted at the 5% significance. All CGM-endpoints will be evaluated on the FAS and CGM-PP population. All other endpoints will be evaluated on the FAS and PP-population. All patients that changed their blood pressure medication during the study will be excluded from the corresponding efficacy analyses.

The following analyses will also be made as exploratory analyses:
-Comparison of CGM-metrics based on PP2 and PP3 where CGM-data are only analysed during the specific days where registration of dietary intake exists
-Correlation analyses for subjects with registration of diet intake in both treatment phases
relating:
A) Difference in amount of carbohydrates with difference in various CGM-metrics between the two treatment phases
B) Difference in the % as carbohydrate constitutes of the total energy intake between the 2 treatment phases with differences of CGM-metrics in the two treatment phases.

The two analyses above (under A and B) will also be performed for blood lipids, blood pressure and weight.

4.8 Safety Analyses

4.8.1 Exposure

Exposure in terms of amount of carbohydrates (E %) in the diet will be summarised descriptively as a continuous variable and categorised into <20E%, 20–30E%, 30–40E%, 40–50E%, 50–60E%, and >60 E% carbohydrates in the diet. Descriptive statistics will be presented per treatment period for the FAS, and PP populations (PP1-PP4). Corresponding descriptive analyses will be performed for the total amount of carbohydrates (gram).

4.8.2 Adverse Events

Number of events and number of patients with events will be presented for the adverse event variables.

5 INTERIM ANALYSES

No interim analysis will be performed.

6 CHANGES OF ANALYSIS FROM PROTOCOL

The following changes have been made before database lock:

- The full analysis set is defined to be all randomised subjects who has registered CGM-data for at least one of the study periods.
- Efficacy analyses will be performed using mixed effects models.

- Missing data will be handled using full maximum likelihood estimation. No imputation is necessary.
- A hierarchical testing procedure will be employed to account for multiple testing of secondary endpoints.

7 LISTING OF TABLE, FIGURES AND LISTINGS

7.1 Listing of Tables

Table Number	Table Title
14.1.1	Patient Disposition and Data Sets Analysed
14.1.2	Protocol Deviations Leading to Exclusion from PP population
14.1.3.1	Demographics and Baseline Characteristics (FAS)
14.1.3.2	Demographics and Baseline Characteristics (PP population)
14.1.4	Medical and Surgical History (FAS)
14.1.5.1	Prior Medications (FAS)
14.1.5.2	Concomitant Medications (FAS)
14.2.1.1	Primary Efficacy Analysis, mean blood glucose level by diet (FAS)
14.2.1.2	Primary Efficacy Analysis, mean blood glucose level by diet (PP population)
14.2.2.1	Secondary Efficacy Analyses by diet (FAS)
14.2.2.2	Secondary Efficacy Analyses by diet (PP population)
14.2.3.1	Exploratory Efficacy Analyses by diet (FAS)
14.2.3.2	Exploratory Efficacy Analyses by diet (PP population)
14.3.1	Exposure by diet (Safety population)
14.3.2.1	Summary of Adverse Events (Safety population)
14.3.2.2	Adverse Events, by Chapter and Diagnosis (Safety population)
14.3.2.3	Severe Adverse Events, by Chapter and Diagnosis (Safety population)
14.3.2.4	Related Adverse Events, by Chapter and Diagnosis (Safety population)
14.3.2.5	Serious Adverse Events, by Chapter and Diagnosis (Safety population)
14.3.3.1	Safety Outcomes by diet (Safety population)
14.3.3.2	Ketone levels by morning/evening and week and diet (Safety population)

7.2 Listing of Figures

Figure Number	Table Title
14.2.1	Mean blood glucose level during run-in, treatment-phases and wash-out.
14.2.2	Standard deviation of blood glucose levels during run-in, treatment-phases and wash-out.
14.2.3	Time above range during run-in, treatment-phases and wash-out.
14.2.4	Time in range during run-in, treatment-phases and wash-out.
14.2.x	To be determined.

7.3 Listing of Listings

Listing number	Listing Title
16.2.1	Discontinued Patients
16.2.2	Patients with Important Protocol Deviations
16.2.3	Patients Excluded from the Efficacy Analysis
16.2.4.1	Demographics and Baseline Characteristics
16.2.4.2	Medical and Surgical History
16.2.4.3	Prior and Concomitant Medications
16.2.4.4	Exposure
16.2.5	Efficacy Variables
16.2.6	Adverse Events

8 REFERENCES

1. Lind M, Polonsky W, Hirsch IB, Heise T, Bolinder J, Dahlqvist S, Schwarz E, Ólafsdóttir AF, Frid A, Wedel H, Ahlén E, Nyström T, Hellman J. Continuous Glucose Monitoring vs Conventional Therapy for Glycemic Control in Adults With Type 1 Diabetes Treated With Multiple Daily Insulin Injections: The GOLD Randomized Clinical Trial. JAMA. 2017 Jan 24;317(4):379-387. doi: 10.1001/jama.2016.19976. Erratum in: JAMA. 2017 May 9;317(18):1912. PMID: 28118454.

9 APPENDIX
