# Supplementary material for: The effect of carbohydrate intake on glycaemic control in individuals with type 1 diabetes: a randomised, open-label, crossover trial
Source: Lancet Reg Health Eur. 2023 Dec 19;37:100799. doi: 10.1016/j.lanepe.2023.100799 (PMC10866914; doi:10.1016/j.lanepe.2023.100799)
Supplement: eTables 1–15 [file mmc3.docx]

**Supplemental appendix**

This supplement has been provided by the authors to provide additional information to their work.

Title: The effect of carbohydrate intake on glycaemic control in individuals with type 1 diabetes: a randomised, open-label, crossover trial

Authors:

Sofia Sterner Isaksson, RD^1,2^, Arndís F. Ólafsdóttir, RN^1,2,3^, Simon Ivarsson, RD^2^, Henrik Imberg, PhD^4,5^, Eva Toft, MD^6,7^, Sara Hallström, MD^1,2,3^, Ulf Rosenqvist, MD^8^, Marie Ekström, RN^2^, Marcus Lind, PhD^1,2,3^

Affiliations:

1. Department of Molecular and Clinical Medicine, Sahlgrenska Academy, University of Gothenburg, Gothenburg, Sweden
2. Department of Medicine, NU Hospital Group, Uddevalla, Sweden
3. Department of Medicine, Sahlgrenska University Hospital, Gothenburg, Sweden
4. Statistiska Konsultgruppen, Gothenburg, Sweden
5. Department of Mathematical Sciences, Chalmers University of Technology and University of Gothenburg, Gothenburg, Sweden
6. Department of Medicine, Ersta Hospital, Stockholm, Sweden
7. Department of Clinical Education and Science, Södersjukhuset, Karolinska Institute, Stockholm, Sweden
8. Department of Internal Medicine, Motala Hospital, Motala, Sweden

**Contents**

**eTable 1.** Inclusion and exclusion criteria for participation in the study.

**eTable 2A–C.** Example of a daily diet plan with moderate carbohydrate diet.

**eTable 3.** Definition of study populations.

**eTable 4.** Baseline characteristics of the per-protocol populations (PP1–PP4).

**eTable 5.** Evaluation of moderate carbohydrate diet vs traditional diet on primary and secondary endpoints in the per-protocol populations.

**eTable 6.** Evaluation of moderate carbohydrate diet vs traditional diet on exploratory endpoints in the FAS population.

**eTable 7.** Evaluation of moderate carbohydrate diet vs traditional diet on exploratory endpoints in the per-protocol populations.

**eTable 8.** Evaluation of moderate carbohydrate diet vs traditional diet on CGM-endpoints during daytime (06:00–21:59) and nighttime (22:00–05:59) in the FAS population.

**eTable 9.** Macronutrients in the diet during moderate carbohydrate diet (MCD) and traditional diet (TD) on the full analysis set (FAS) and per-protocol populations (PP2–PP3).

**eTable 10.** Summary of adverse events (safety population).

**eTable 11.** Adverse events (safety population).

**eTable 12.** Medical and surgical history (Full Analysis Set).

**eTable 13.** Prior Medications (Full Analysis Set)**.**

**eTable 14.** Concomitant Medications (Full Analysis Set)**.**

**eTable 15.** Post-hoc analysis of mean glucose measured by the area under the curve (AUC) of glucose levels measured by CGM during moderate carbohydrate diet and traditional diet on the full analysis set (FAS) and per-protocol populations (PP1–PP4).

**eTable 1. Inclusion and exclusion criteria for participation in the study.**

| **Inclusion criteria** |
| --- |
| Type 1 diabetes |
| Adults 18 years or older |
| Written informed consent |
| HbA1c ≥ 58 mmol/mol (7.5% DCCT standard) |
| **Exclusion criteria** |
| Pregnancy or planned pregnancy during the study period |
| Severe cognitive dysfunction or other disease as determined by the physician. |
| Inability to comply with study diet (excluding foods common to each diet such as wholegrains, beans, lentils, fruit, and vegetables because of personal preferences) |
| Other disease |
| Diabetes duration < 1 year |
| Planned change in diabetes treatment (e.g., commencing insulin pump or CGM) during the study period. |

**eTable 2A–C. Example of a daily diet plan with moderate carbohydrate diet**

This example is calculated to fit a person with a daily energy intake of 2500 kcal and a daily intake of approximately 200-gram carbohydrates and 30 percent of energy from carbohydrates. All participants also received recipes, and a detailed description of what to add and reduce in the current diet to be able to reach the macronutrient goals of the diet as well as include a lot of healthy foods providing minerals, vitamins, and fibres. This example has been translated from Swedish.

**eTable 2A. Breakfast**

Select **one** alternative for breakfast. All alternatives include approximately 40 g carbohydrates and 500 kcal each.

| 1. Yoghurt breakfast   3 dl plain yoghurt 3 % fat  0.5 dl oats  2 dl berries (raspberries/blueberries etc.)  30 g nuts/seeds/almonds  0.5 teaspoon flaxseed | 1. Oatmeal breakfast   35 g (1 dl) oats (cook the porridge with water according to instructions on the package)  30 g nuts/seeds/almonds  0.5 teaspoon flaxseed  ½ medium sized apple, chopped  2 dl milk (1.5 % fat)  1 boiled egg |
| --- | --- |
| 1. Sandwich breakfast   2 slices (ca 70 g) of wholegrain bread  15 g sandwich margarine  60 g sliced turkey  30 g cheese (28 % fat)  Vegetables, e.g., pepper/salad/cucumber/tomato  ½ grapefruit | 1. Crisp bread breakfast   4 slices crisp bread á 12 g per piece  15 g sandwich margarine  60 g sliced turkey  30 g cheese (28 % fat)  Vegetables, e.g., pepper/salad/cucumber/tomato  ½ grapefruit |

**eTable 2B. Lunch and dinner**

Eat this type of meal 2 times per day (lunch and dinner) and select among the alternatives and include something from 1, 2, 3 and 4 below in the meal. All alternatives contain approximately 50-gram carbohydrates and 600 kcal.

| 1. 220 g potatoes ***or***   175 g boiled” whole wheat”/” whole grains”/whole oats”/bulgur/quinoa (55 g unboiled) ***or***  145 g boiled rice (50 g unboiled) ***or***  135 g boiled pasta (50 g unboiled) | | |
| --- | --- | --- |
| 1. 110 g lean meat. E.g., lean fish, chicken/turkey without skin, lean ham, or venison   **or**  110 g meat containing more fat. E.g.,  salmon, herring, chicken including skin, sausage/meatballs | +  + | 2 tablespoon oil ***or***  2.5 tablespoon mayonnaise/liquid margarine/fat (80%) ***or*** 80 g crème fraiche (34%)  1 tablespoon oil ***or***  1.5 tablespoon mayonnaise/liquid margarine/fat (80%) ***or***  50 g crème fraiche (34 %) ***or***  100 g crème fraiche light (15 %) |
| 1. 40 g chickpeas/kidney beans/white beans/black beans etc. | | |
| 1. Free amount of vegetables, preferably >150 g | | |

**eTable 2C. Snacks**

Eat 4 of these per day and feel free to combine 2-3 of them in one snack. All of them contains approximately 12-15 grams carbohydrates and 200 kcal.

| 1. 80 g Hummus (se provided recipe) + cucumber/pepper/cabbage in pieces/sticks (free amount) |
| --- |
| 1. 20 g raisins + 25 g peanuts |
| 1. 50 g quark (1 % fat) mixed with 50 g plain yoghurt (3 % fat) + ½ chopped apple + 2 tablespoons sunflower seeds/chopped nuts/almonds + cinnamon/cardamom for extra flavour. |
| 1. 100 g cottage cheese (4 % fat) + 100 g chopped fruit (e.g., pear/apple/kiwi/nectarine) + 2 teaspoons sunflower seeds/chopped nuts/almonds + cinnamon/cardamom for extra flavour. |
| 1. Sandwiches: 18 g crisp bread/”Finncrisp” ***or*** 25 g wholegrain bread with ***ONE*** of the following toppings: |
| 1. ½ can mackerel in tomato sauce (65 g). 2. 50 g cottage cheese (4 % fat) mixed with 2 teaspoons pesto. 3. 1 boiled egg + 5 g Flora margarine + 10 g caviar 4. 45 g smoked salmon + 30 g cream cheese (e.g., Philadelphia cheese) 5. 2 slices smoked turkey/ham + ½ avocado |

**eTable 3. Definition of study populations.**

| **Study population** | **Definition** |
| --- | --- |
| **Full Analysis Set** | All randomised subjects with CGM-data^a^ available for at least one of the treatment phases. |
| **PP1** | All subjects in the FAS who have no protocol deviations indicating that they have not received diet advise or complied with the diets and who have measurements of CGM in both treatment phases (including eCRF data) within a time-period not significantly deviating from the planned time-period. |
| **PP2** | All subjects in PP1 who in addition have diet records in both treatment phases confirming that the total amount of carbohydrates deviated between the treatment phases with a lower amount during the treatment sequence aimed at reducing the amount of carbohydrates. |
| **PP3** | All subjects in PP2 that in addition have a lower % of the total energy intake constituted of carbohydrates during the treatment sequence with moderately reduced carbohydrate intake. |
| **PP4 (CGM-PP population)** | All subjects in the PP1-population with at least 9 days with CGM data for least 70% of the time, in both diet periods. |
| **Safety population** | All randomised subjects who received the diet with moderate amount of carbohydrates during any time period. In the safety analysis a subject will belong to the treatment given not to the randomised treatment. |
| ^a^ At least 3 days with CGM data for at least 70% of the time, or at least 3 days of CGM data in total (from blinded CGM or from the participants own CGM device), or CGM mean recorded in the eCRF. | |

**eTable 4. Baseline characteristics of the per-protocol populations (PP1–PP4).**

| **Variable** | **PP1 (n=43)** | **PP2 (n=27)** | **PP3 (n=20)** | **PP4 (n=33)** |
| --- | --- | --- | --- | --- |
| **Age (years)** | 48.4 (13.8) 49 (23–73) | 47.6 (13.0) 49 (26–73) | 46.2 (13.1) 48.5 (26–64) | 51.5 (13.2) 53 (27–73) |
| **Female sex** | 21 (48.8%) | 15 (55.6%) | 12 (60.0%) | 19 (57.6%) |
| **Diabetes duration (years)** | 25.5 (12.5) 26 (2–54) | 26.1 (11.8) 26 (2–54) | 26.1 (12.9) 26 (2–54) | 26.8 (12.5) 27 (8–54) |
| **Haemoglobin A1c (mmol/mol)** | 68.2 (11.1) 64 (58–105) | 66.7 (8.5) 63 (58–86) | 65.9 (8.5) 62.5 (58–86) | 66.4 (9.0) 63 (58–96) |
| **Haemoglobin A1c (%)** | 8.39 (1.01) 8.01 (7.46–11.76) | 8.25 (0.78) 7.91 (7.46–10.02) | 8.18 (0.78) 7.87 (7.46–10.02) | 8.23 (0.83) 7.91 (7.46–10.93) |
| **Weight (kg)** | 88.0 (16.5) 85 (60–130) | 88.0 (16.9) 84 (60–130) | 90.1 (18.9) 86 (60–130) | 85.3 (15.4) 83 (60–127) |
| **Body mass index (kg/m^2^)** | 29.5 (4.7) 29.1 (21.5–47.8) | 29.5 (4.4) 28.1 (24–43.8) | 29.9 (4.9) 28.7 (24–43.8) | 29.3 (4.8) 29.3 (21.5–47.8) |
| **Smoking** | 3 (7.0%) | 3 (11.1%) | 3 (15.0%) | 3 (9.1%) |
| **CGM mean (mmol/L)** | 9.15 (2.22) 8.99 (4.53–14.12) | 9.22 (2.41) 9.03 (4.53–14.12) | 9.46 (2.53) 9.69 (4.53–14.12) | 8.75 (2.10) 8.22 (4.53–12.4) |
| **Percent time in range 3.9–10 mmol/L** | 53.2 (15.8) 49.4 (22.8–81.2) | 50.6 (15.3) 48.1 (22.8–81.2) | 48.5 (15.3) 48.1 (22.8–81.2) | 55.5 (15.5) 52.9 (29–81.2) |
| **Percent time above range >10 mmol/L** | 38.2 (20.0) 37.7 (4.4–77.2) | 39.5 (20.6) 38.7 (4.4–77.2) | 41.3 (21.9) 43.3 (4.4–77.2) | 35.0 (19.6) 33.7 (4.4–68) |
| **Percent time in hyperglycaemia >13.9 mmol/L** | 13.8 (12.3) 11.7 (0–46.1) | 15.1 (13.2) 13.8 (0–46.1) | 17.0 (14.0) 15 (0–46.1) | 11.0 (10.6) 8.9 (0–36.7) |
| **Percent time in hypoglycaemia <3.0 mmol/L** | 3.51 (4.87) 1.62 (–20.2) | 4.02 (5.19) 2.18 (0–20.2) | 4.45 (5.27) 2.56 (0–20.2) | 3.76 (4.94) 2.18 (0–20.2) |
| **Percent time below range <3.9 mmol/L** | 8.59 (9.27) 5.49 (0–44.78) | 9.95 (10.24) 6.12 (0–44.78) | 10.2 (11.4) 6.1 (0–44.8) | 9.42 (9.89) 6.12 (0–44.78) |
| **Systolic blood pressure (mmHg)** | 132.4 (13.8) 131 (90–160) | 133.1 (13.5) 131 (101–160) | 133.9 (14.1) 131.5 (101–160) | 133.4 (13.7) 131 (90–160) |
| **Diastolic blood pressure (mmHg)** | 74.1 (9.1) 76 (55–96) | 76.2 (10.2) 78 (60–103) | 76.6 (11.3) 76.5 (60–103) | 72.2 (8.5) 74 (55–87) |
| **Total cholesterol (mmol/L)** | 4.07 (0.77) 4.2 (2.7–6.1) | 4.19 (0.75) 4.2 (2.8–6.1) | 4.18 (0.84) 4.2 (2.8–6.1) | 4.05 (0.69) 4.2 (2.7–5.4) |
| **Low-density lipoprotein (mmol/L)** | 2.23 (0.67) 2.1 (1–4.1) | 2.27 (0.59) 2.1 (1.5–4) | 2.30 (0.67) 2.1 (1.5–4) | 2.16 (0.61) 2.1 (1–4.1) |
| **High-density lipoprotein (mmol/L)** | 1.45 (0.45) 1.4 (0.73–2.6) | 1.51 (0.42) 1.5 (0.83–2.4) | 1.46 (0.39) 1.45 (0.83–2.4) | 1.52 (0.44) 1.5 (0.73–2.6) |
| **Triglycerides (mmol/L)** | 1.20 (0.64) 1.1 (0.4–2.8) | 1.22 (0.64) 1.1 (0.52–2.8) | 1.29 (0.61) 1.15 (0.63–2.8) | 1.09 (0.53) 1 (0.4–2.4) |
| **Insulin pump** | 15 (34.9%) | 8 (29.6%) | 5 (25.0%) | 8 (24.2%) |
| **Total daily insulin dose (IU)^b^** | 60.3 (38.3) 47.5 (23.9–206) | 57.2 (31.1) 47.5 (23.9–167.3) | 61.5 (33.1) 50 (25.8–167.3) | 56.5 (29.3) 47.4 (25.8–167.3) |
| **Daily mealtime insulin (IU) ^b^** | 24.3 (18.8) 20.4 (5.8–91) | 23.5 (18.0) 17.3 (5.8–91) | 26.4 (19.7) 21.9 (8.3–91) | 23.1 (16.9) 18.9 (5.8–91) |
| **Daily basal insulin (IU)^b^** | 35.0 (22.4) 28 (11–136) | 32.7 (15.6) 28 (11–76.3) | 34.0 (16.1) 31.5 (11–76.3) | 32.2 (14.9) 27 (11–76.3) |
| **Total daily insulin dose (IU) ^b^** | 60.3 (38.3) 47.5 (23.9–206) | 57.2 (31.1) 47.5 (23.9–167.3) | 61.5 (33.1) 50 (25.8–167.3) | 56.5 (29.3) 47.4 (25.8–167.3) |
| Data are presented as mean (standard deviation), median (minimum–maximum value) for numeric variables and as numbers and percentages for categorical variables. | | | | |

**eTable 5. Evaluation of moderate carbohydrate diet vs traditional diet on primary and secondary endpoints in the per-protocol populations**.

| **Variable** | **PP1**  **(n=43)** | | | **PP2**  **(n=27)** | | | **PP3**  **(n=20)** | | | **PP4**  **(n=33)** | | |
| --- | --- | --- | --- | --- | --- | --- | --- | --- | --- | --- | --- | --- |
|  | **MCD** | **TD** | **Mean diff /**  **Fold change**  **(95% CI)** | **MCD** | **TD** | **Mean diff /**  **Fold change**  **(95% CI)** | **MCD** | **TD** | **Mean diff /**  **Fold change**  **(95% CI)** | **MCD** | **TD** | **Mean diff/**  **Fold change**  **(95% CI)** |
| **CGM mean (mmol/L)** | 8.6 (1.6) | 9.1 (1.7) | -0.5  (-0.9 to -0.2) p=0.003 | 8.2 (1.4) | 8.9 (1.6) | -0.7  (-1.1 to -0.2) p=0.010 | 8.2 (1.3) | 8.7 (1.7) | -0.6  (-1.2 to 0.1) p=0.080 | 8.2 (1.4) | 8.7 (1.5) | -0.6  (-1.0 to-0.2) p=0.005 |
| **CGM SD (mmol/L)** | 3.6 (1.0) | 3.7 (1.0) | -0.1  (-0.3 to 0.1) p=0.28 | 3.4 (0.7) | 3.5 (0.8) | -0.2  (-0.4 to 0.1) p=0.12 | 3.3 (0.7) | 3.4 (0.8) | -0.1  (-0.4 to 0.2) p=0.33 | 3.3 (0.7) | 3.4 (0.6) | -0.1  (-0.3 to 0.1) p=0.22 |
| **Percent time in range 3.9–10 mmol/L** | 58.2 (12.5) | 54.3 (15.9) | 4.2  (0.5 to 7.8) p=0.027 | 61.6 (12.1) | 56.7 (16.4) | 5.1  (-0.2 to 10.5) p=0.060 | 61.9 (11.5) | 58.6 (17.0) | 3.6  (-3.2 to 10.4) p=0.28 | 61.1 (11.0) | 57.8 (15.1) | 3.5  (-0.8 to 7.9) p=0.11 |
| **Percent time above range >10 mmol/L** | 32.0 (15.7) | 37.3 (18.4) | -5.6  (-9.6 to -1.5) p=0.008 | 28.5 (15.6) | 34.9 (19.0) | -6.6  (-12.7 to -0.5) p=0.035 | 28.1 (15.1) | 33.0 (19.7) | -5.2  (-13.1 to 2.7) p=0.18 | 28.3 (14.8) | 33.7 (17.9) | -5.7  (-10.5 to -0.9) p=0.022 |
| **Percent time in hyperglycaemia >13.9 mmol/L** | 10.0 (9.7) | 12.6 (10.5) | -2.7  (-4.9 to -0.5) p=0.016 | 8.4 (7.4) | 11.4 (10.0) | -3.2  (-6.6 to 0.2) p=0.061 | 7.5 (5.4) | 10.1 (10.5) | -2.7  (-7.0 to 1.5) p=0.19 | 7.1 (6.9) | 9.5 (8.0) | -2.6  (-4.9 to -0.2) p=0.034 |
| **Percent time in hypoglycaemia <3.0 mmol/L** | 4.0 (4.2) | 3.2 (3.3) | 0.8  (-0.3 to 1.8) p=0.14 | 3.5 (3.3) | 3.5 (3.7) | -0.1  (-1.6 to 1.4) p=0.92 | 3.3 (2.8) | 3.7 (3.3) | -0.4  (-2.4 to 1.6) p=0.67 | 4.3 (4.4) | 3.3 (3.4) | 1.0  (-0.4 to 2.3) p=0.14 |
| **Percent time below range <3.9 mmol/L** | 9.8 (7.5) | 8.4 (6.2) | 1.4  (-0.4 to 3.2) p=0.11 | 10.0 (7.2) | 8.5 (6.5) | 1.5  (-1.3 to 4.3) p=0.27 | 10.0 (7.3) | 8.4 (6.0) | 1.6  (-2.2 to 5.4) p=0.38 | 10.6 (8.1) | 8.4 (6.3) | 2.2  (0.0 to 4.3) p=0.048 |
| **Weight (kg)** | 87.2 (16.9) | 87.5 (16.8) | -0.1  (-0.5 to 0.3) p=0.54 | 87.1 (16.7) | 87.3 (16.9) | -0.2  (-0.6 to 0.2) p=0.36 | 89.5 (18.6) | 89.7 (18.7) | -0.2  (-0.7 to 0.3) p=0.41 | 84.3 (15.2) | 84.5 (15.2) | -0.2  (-0.6 to 0.2) p=0.29 |
| **Total cholesterol (mmol/L) ^a^** | 4.0  (3.5–4.5) | 4.0  (3.5–4.7) | 0.99  (0.95 to 1.02)  p=0.38 | 4.1  (3.7–4.6) | 4.2  (3.5–4.8) | 1.01  (0.97 to 1.05) p=0.78 | 4.1  (3.6–4.6) | 4.1  (3.6– 4.8) | 1.00  (0.95 to 1.04) p=0.85 | 4.1  (3.4– 4.5) | 4.0  (3.4– 4.5) | 1.00  (0.96 to 1.04) p=0.87 |
| **Low-density lipoprotein (mmol/L) ^a^** | 2.1  (1.6–2.6) | 2.1  (1.7–2.5) | 0.98  (0.92 to 1.04) p=0.52 | 2.2  (1.6–2.7) | 2.2  (1.8– 2.5) | 0.98  (0.91 to 1.06) p=0.63 | 2.2  (1.6–2.8) | 2.2  (1.9–2.6) | 0.99  (0.90 to 1.09) p=0.76 | 2.0  (1.5–2.4) | 2.0  (1.7–2.4) | 1.00  (0.93 to 1.08) p=0.98 |
| **High-density lipoprotein (mmol/L) ^a^** | 1.4  (1.1–1.8) | 1.4  (1.1–1.7) | 1.00  (0.95 to 1.05) p=0.92 | 1.5  (1.2–1.8) | 1.4  (1.1–1.8) | 1.04  (0.98 to 1.11) p=0.15 | 1.4  (1.1–1.8) | 1.4  (1.2– 1.6) | 1.01  (0.95 to 1.06) p=0.84 | 1.5  (1.2–1.8) | 1.5  (1.3– 1.8) | 1.01  (0.95 to 1.07) p=0.79 |
| **Triglycerides (mmol/L) ^a^** | 0.8  (0.6–1.3) | 0.9  (0.7–1.3) | 0.95  (0.86 to 1.06) p=0.39 | 0.8  (0.6– 1.4) | 0.9  (0.7–1.2) | 1.01  (0.89 to 1.15) p=0.85 | 0.9  (0.7– 1.3) | 0.9  (0.7–1.3) | 1.06  (0.92 to 1.23) p=0.39 | 0.8  (0.6– 1.0) | 0.9  (0.7–1.2) | 0.95  (0.85 to 1.07) p=0.40 |
| **Total daily insulin dose (IU)** | 56.8 (40.9) | 61.2 (40.2) | -4.0  (-8.3 to 0.3) p=0.067 | 50.9 (30.9) | 57.2 (31.9) | -6.2  (-10.9 to -1.4) p=0.013 | 53.1 (32.2) | 60.5 (33.1) | -7.2  (-13.5 to -1.0) p=0.026 | 50.3 (29.8) | 55.9 (31.2) | -4.6  (-8.0 to -1.1) p=0.011 |
| **DTSQs total score** | 28.9 (5.1) | 27.8 (5.8) | 1.3  (-0.1 to 2.6) p=0.065 | 28.9 (5.2) | 27.4 (6.2) | 1.4  (-0.5 to 3.4) p=0.15 | 29.5 (5.5) | 28.2 (6.3) | 1.3  (-0.9 to 3.5) p=0.24 | 30.2 (4.8) | 28.5 (5.4) | 1.7  (0.4 to 3.0) p=0.013 |
| **Hypoglycemia confidence scale mean score** | 3.5 (0.5) | 3.4 (0.4) | 0.1  (-0.0 to 0.2) p=0.18 | 3.6 (0.5) | 3.5 (0.5) | 0.1  (-0.1 to 0.2) p=0.37 | 3.6 (0.5) | 3.6 (0.4) | 0.0  (-0.1 to 0.1) p=0.77 | 3.5 (0.5) | 3.4 (0.4) | 0.1  (-0.1 to 0.2) p=0.38 |
| Data are presented as mean (SD) and mean difference (95% CI) or median (IQR) and fold-change (95% CI).  Statistical analyses were performed using linear mixed effects models with treatment (diet) and period as fixed effects and subject as random effect.  ^a^Fold change between groups is presented.  **Abbreviations:** CI, confidence interval; IQR, interquartile range; MCD, moderate carbohydrate diet; PP, per-protocol; SD, standard deviation; TD, traditional diet. | | | | | | | | | | | | |

**eTable 6. Evaluation of moderate carbohydrate diet vs traditional diet on exploratory endpoints in the FAS population.**

| **Variable** | **Moderate carbohydrate diet** | **Traditional diet** | **Mean difference**  **/ Fold change**  **(95% CI)** |
| --- | --- | --- | --- |
| Percent time in tight range (3.9–7.8 mmol/L) | 38.6 (13.5) | 34.3 (14.8) | 4.5 (1.3 to 7.8) p*=*0.008 |
| Percent time in target  (3.5–7.8 mmol/L) | 41.2 (14.8) | 36.6 (15.8) | 4.8 (1.2 to 8.4) p=0.010 |
| MAGE (mmol/L) | 8.6 (2.2) | 8.8 (1.6) | -0.3 (-0.7 to 0.2) p=0.26 |
| CV (%) | 41.5 (7.5) | 40.8 (8.6) | 0.7 (-1.3 to 2.7) p=0.50 |
| Haemoglobin A1c (mmol/mol) | 62.2 (9.6) | 63.1 (11.8) | -1.1 (-2.7 to 0.5) p=0.18 |
| Systolic blood pressure (mmHg) | 126.9 (13.3) | 129.1 (12.7) | -2.7 (-6.1 to 0.7) p=0.11 |
| Diastolic blood pressure (mmHg) | 71.6 (8.6) | 72.4 (8.5) | -1.0 (-3.1 to 1.1) p=0.33 |
| Apolipoprotein A (g/L) ^a^ | 1.54 (1.34–1.76) | 1.60 (1.39–1.78) | 0.98 (0.95 to 1.01) p=0.16 |
| Apolipoprotein B (g/L) ^a^ | 0.77 (0.64–0.84) | 0.76 (0.65–0.88) | 0.97 (0.93 to 1.01) p=0.17 |
| Apolipoprotein A/B ratio ^a^ | 0.47 (0.40–0.59) | 0.46 (0.40–0.59) | 0.99 (0.95 to 1.04) p=0.76 |
| Daily mealtime insulin (IU) | 22.8 (19.1) | 23.5 (17.5) | -1.0 (-3.8 to 1.8) p=0.49 |
| Daily basal insulin (IU) | 34.7 (23.7) | 35.9 (23.3) | -1.1 (-2.8 to 0.7) p=0.24 |
| Total daily insulin dose to body weight ratio (IU/kg) | 0.6 (0.3) | 0.7 (0.3) | -0.0 (-0.1 to 0.0) p=0.054 |
| Data are presented as mean (SD) and mean difference (95% CI) or median (IQR) and fold-change (95% CI).  Statistical analyses were performed using linear mixed effects models with treatment (diet) and period as fixed effects and subject as random effect.  ^a^Fold change between groups is presented.  **Abbreviations:** CI, confidence interval; CV, coefficient of variation; IQR, interquartile range; MAGE, mean amplitude of glycaemic excursions; SD, standard deviation. | | | |

**eTable 7. Evaluation of moderate carbohydrate diet vs traditional diet on exploratory endpoints in the per-protocol populations.**

| **Variable** | **PP1**  **(n=43)** | | | **PP2**  **(n=27)** | | | **PP3**  **(n=20)** | | | **PP4**  **(n=33)** | | |
| --- | --- | --- | --- | --- | --- | --- | --- | --- | --- | --- | --- | --- |
|  | **MCD** | **TD** | **Mean diff /**  **Fold change**  **(95% CI)** | **MCD** | **TD** | **Mean diff /**  **Fold change**  **(95% CI)** | **MCD** | **TD** | **Mean diff /**  **Fold change**  **(95% CI)** | **MCD** | **TD** | **Mean diff/**  **Fold change**  **(95% CI)** |
| **Percent time in tight range (3.9–7.8 mmol/L)** | 38.7 (13.0) | 34.6 (14.5) | 4.4  (0.8 to 8.0) p=0.018 | 42.6 (13.8) | 36.5 (16.0) | 6.4  (1.1 to 11.7) p=0.020 | 43.1 (14.6) | 38.6 (17.2) | 4.7  (-2.4 to 11.7) p=0.18 | 41.3 (13.0) | 37.0 (14.7) | 4.6  (0.4 to 8.9) p=0.032 |
| **Percent time in target**  **(3.5–7.8 mmol/L)** | 41.3 (14.3) | 37.0 (15.5) | 4.6  (0.7 to 8.6) p=0.024 | 45.7 (15.2) | 38.8 (16.9) | 7.1  (1.4 to 12.8) p=0.016 | 46.1 (16.1) | 40.8 (18.1) | 5.6  (-2.1 to 13.3) p=0.14 | 44.2 (14.5) | 39.4 (15.8) | 5.1  (0.5 to 9.7) p=0.032 |
| **MAGE (mmol/L)** | 8.6 (2.3) | 8.7 (1.7) | -0.2  (-0.7 to 0.3) p=0.47 | 8.0 (1.7) | 8.4 (1.6) | -0.4  (-0.9 to 0.1) p=0.14 | 7.9 (1.7) | 8.1 (1.7) | -0.2  (-0.8 to 0.3) p=0.39 | 7.8 (1.6) | 8.1 (1.3) | -0.4  (-0.8 to 0.1) p=0.12 |
| **CV (%)** | 41.9 (7.6) | 40.9 (8.6) | 0.9  (-1.2 to 3.1) p=0.38 | 40.9 (5.9) | 40.3 (8.1) | 0.6  (-2.0 to 3.3) p=0.62 | 40.2 (5.2) | 39.4 (7.2) | 0.8  (-2.7 to 4.2) p=0.65 | 40.7 (7.1) | 39.5 (7.2) | 1.2  (-1.1 to 3.4) p=0.31 |
| **Haemoglobin A1c (mmol/mol)** | 60.9 (8.5) | 61.5 (10.4) | -0.6  (-2.3 to 1.0) p=0.43 | 59.2 (6.0) | 59.8 (7.7) | -0.6  (-2.8 to 1.5) p=0.54 | 58.9 (4.9) | 59.1 (8.0) | -0.1  (-2.5 to 2.2) p=0.89 | 60.0 (7.1) | 59.6 (8.2) | 0.4  (-1.1 to 2.0) p=0.55 |
| **Systolic blood pressure (mmHg)** | 126.4 (13.1) | 129.8 (11.4) | -3.5  (-7.1 to 0.0) p=0.052 | 125.1 (12.6) | 128.0 (10.1) | -2.7  (-6.6 to 1.1) p=0.16 | 124.0 (12.9) | 127.0 (9.4) | -3.0  (-7.1 to 1.1) p=0.14 | 126.9 (14.7) | 130.9 (12.0) | -4.1  (-8.3 to 0.2) p=0.061 |
| **Diastolic blood pressure (mmHg)** | 71.0 (8.4) | 72.1 (8.7) | -1.3  (-3.7 to 1.1) p=0.27 | 72.2 (9.3) | 71.9 (8.4) | 0.4  (-2.5 to 3.4) p=0.76 | 72.2 (9.7) | 70.9 (8.3) | 1.3  (-2.1 to 4.7) p=0.43 | 69.5 (8.4) | 70.4 (8.5) | -1.0  (-3.7 to 1.7) p=0.46 |
| **Apolipoprotein A (g/L) ^a^** | 1.54  (1.31–1.73) | 1.55  (1.36–1.78) | 0.98  (0.95 to 1.01) p=0.19 | 1.55  (1.48–1.80) | 1.60  (1.37–1.91) | 0.99  (0.95 to 1.04) p=0.80 | 1.53  (1.32–1.66) | 1.58  (1.38–1.74) | 0.98  (0.94 to 1.01) p=0.19 | 1.57  (1.48–1.78) | 1.60  (1.40–1.80) | 0.98  (0.95 to 1.02) p=0.33 |
| **Apolipoprotein B (g/L) ^a^** | 0.71  (0.63–0.84) | 0.73  (0.65–0.86) | 0.98  (0.94 to 1.02) p=0.31 | 0.80  (0.64–0.87) | 0.75  (0.65–0.83) | 0.99  (0.94 to 1.04) p=0.67 | 0.76  (0.66–0.85) | 0.76  (0.66–0.81) | 0.99  (0.93 to 1.06) p=0.84 | 0.71  (0.64–0.83) | 0.70  (0.64–0.80) | 1.00  (0.95 to 1.05) p=0.88 |
| **Apolipoprotein A/B ratio ^a^** | 0.45  (0.39–0.59) | 0.44  (0.39–0.59) | 1.00  (0.95 to 1.05) p=0.96 | 0.45  (0.41–0.55) | 0.46  (0.39–0.58) | 0.99  (0.93 to 1.06) p=0.85 | 0.46  (0.41–0.56) | 0.45  (0.40–0.57) | 1.02  (0.95 to 1.09) p=0.59 | 0.45  (0.39–0.55) | 0.44  (0.39–0.56) | 1.01  (0.95 to 1.08) p=0.65 |
| **Daily mealtime insulin (IU)** | 22.7 (19.6) | 24.3 (18.0) | -1.5  (-4.3 to 1.3) p=0.29 | 21.3 (18.4) | 23.6 (16.9) | -2.5  (-5.3 to 0.2) p=0.067 | 22.4 (19.9) | 24.7 (17.9) | -2.9  (-6.4 to 0.6) p=0.10 | 20.5 (16.8) | 23.5 (16.1) | -2.5  (-4.8 to -0.3) p=0.029 |
| **Daily basal insulin (IU)** | 35.3 (24.5) | 36.6 (24.0) | -1.3  (-3.2 to 0.7) p=0.19 | 31.9 (16.0) | 33.9 (16.9) | -1.9  (-4.3 to 0.4) p=0.10 | 32.7 (16.2) | 34.9 (17.5) | -2.1  (-5.3 to 1.0) p=0.17 | 29.9 (15.3) | 32.3 (16.4) | -2.0  (-4.0 to 0.0) p=0.054 |
| **Total daily insulin dose to body weight ratio (IU/kg)** | 0.6 (0.3) | 0.7 (0.3) | -0.0  (-0.1 to -0.0) p=0.033 | 0.6 (0.3) | 0.6 (0.3) | -0.1  (-0.1 to -0.0) p=0.006 | 0.6 (0.3) | 0.7 (0.3) | -0.1  (-0.1 to -0.0) p=0.013 | 0.6 (0.3) | 0.6 (0.3) | -0.0  (-0.1 to -0.0) p=0.008 |
| Data are presented as mean (SD) and mean difference (95% CI) or median (IQR) and fold-change (95% CI).  Statistical analyses were performed using linear mixed effects models with treatment (diet) and period as fixed effects and subject as random effect.  ^a^Fold change between groups is presented.  **Abbreviations:** CI, confidence interval; CV, coefficient of variations; IQR, interquartile range; MAGE, mean amplitude of glycaemic excursions; MCD, moderate carbohydrate diet; PP, per-protocol; SD, standard deviation; TD, traditional diet. | | | | | | | | | | | | |

**eTable 8**. **Evaluation of moderate carbohydrate diet vs traditional diet on CGM-endpoints during daytime (06:00–21:59) and nighttime (22:00–05:59) in the FAS population.**

| **Variable** | **Daytime** | | | **Nighttime** | | |
| --- | --- | --- | --- | --- | --- | --- |
|  | **Moderate carbohydrate diet** | **Traditional diet** | **Mean difference**  **(95% CI)** | **Moderate carbohydrate diet** | **Traditional diet** | **Mean difference**  **(95% CI)** |
| **CGM mean (mmol/L)** | 8.6 (1.8) | 9.3 (1.9) | -0.7 (-1.0 to -0.3) p*<*0.001 | 8.8 (1.9) | 9.2 (2.3) | -0.5 (-1.0 to 0.1) p=0.078 |
| **CGM SD (mmol/L)** | 3.5 (1.0) | 3.7 (1.0) | -0.2 (-0.5 to -0.0) p=0.023 | 3.6 (1.2) | 3.6 (1.0) | 0.1 (-0.2 to 0.3) p=0.54 |
| **Percent time in range 3.9–10 mmol/L** | 59.0 (14.1) | 54.4 (17.1) | 4.9 (1.2 to 8.5) p=0.010 | 55.6 (16.5) | 51.7 (19.7) | 4.2 (-0.5 to 8.9) p=0.076 |
| **Percent time above range >10 mmol/L** | 32.0 (17.4) | 38.1 (19.2) | -6.4 (-10.0 to -2.9) p<0.001 | 34.2 (18.6) | 38.9 (23.6) | -5.0 (-10.4 to 0.4) p=0.070 |
| **Percent time in hyperglycaemia >13.9 mmol/L** | 9.7 (10.6) | 14.2 (14.0) | -4.7 (-7.2 to -2.3) p<0.001 | 11.6 (11.5) | 12.7 (12.9) | -1.4 (-4.9 to 2.1) p=0.43 |
| **Percent time in hypoglycaemia <3.0 mmol/L** | 3.3 (3.7) | 2.6 (2.7) | 0.7 (-0.4 to 1.8) p=0.23 | 4.8 (6.0) | 4.3 (5.3) | 0.5 (-1.2 to 2.2) p=0.57 |
| **Percent time below range <3.9 mmol/L** | 9.0 (7.3) | 7.5 (5.5) | 1.6 (-0.2 to 3.4) p=0.086 | 10.2 (8.9) | 9.4 (9.1) | 0.7 (-1.8 to 3.3) p=0.56 |
| **Percent time in tight range (3.9–7.8 mmol/L)** | 39.2 (13.9) | 35.3 (15.1) | 4.1 (0.9 to 7.4) p=0.015 | 37.2 (16.1) | 32.4 (17.2) | 5.3 (0.7 to 9.8) p=0.025 |
| **Percent time in target (3.5–7.8 mmol/L)** | 41.9 (15.4) | 37.6 (16.2) | 4.5 (1.0 to 8.0) p=0.012 | 39.7 (17.0) | 34.8 (18.3) | 5.3 (0.3 to 10.3) p=0.039 |
| **MAGE (mmol/L)** | 7.6 (2.0) | 8.0 (1.6) | -0.4 (-0.8 to 0.1) p=0.084 | 6.8 (2.8) | 6.9 (2.0) | -0.1 (-1.0 to 0.9) p=0.91 |
| **CV (%)** | 40.6 (8.2) | 40.4 (8.3) | 0.2 (-2.0 to 2.4) p=0.087 | 41.3 (8.6) | 39.8 (11.6) | 1.5 (-1.3 to 4.4) p=0.29 |
| Data are presented as mean (SD) and mean difference (95% CI) or median (IQR) and fold-change (95% CI).  Statistical analyses were performed using linear mixed effects models with treatment (diet) and period as fixed effects and subject as random effect.  **Abbreviations:** CI, confidence interval; CV, coefficient of variations; IQR, interquartile range; MAGE, mean amplitude of glycaemic excursions; SD, standard deviation. | | | | | | |

**eTable 9. Macronutrients in the diet during moderate carbohydrate diet (MCD) and traditional diet (TD) on the full analysis set (FAS) and per-protocol populations (PP2–PP3).**

|  | **Total amount of**  **carbohydrates** | | **Total amount of fat** | | **Total amount of protein** | |
| --- | --- | --- | --- | --- | --- | --- |
| Study Population / Diet | g | E % | g | E % | g | E % |
| **FAS** |  |  |  |  |  |  |
| MCD (n=47) | 155 (40.2) | 34.2 (5.7) | 90.0 (26.4) | 43.4 (6.5) | 82.0 (20.3) | 18.2 (3.2) |
| TD (n=41) | 187 (50.7) | 40.5 (6.9) | 80.5 (30.3) | 37.4 (7.4) | 80.8 (27.2) | 17.2 (3.4) |
| **PP2** |  |  |  |  |  |  |
| MCD (n=27) | 145 (30.8) | 32.7 (5.8) | 92.0 (28.5) | 44.1 (6.5) | 84.2 (19.7) | 18.7 (3.1) |
| TD (n=27) | 199 (51.8) | 41.4 (7.8) | 82.4 (34.2) | 35.9 (7.7) | 86.6 (29.4) | 17.5 (3.7) |
| **PP3** |  |  |  |  |  |  |
| MCD (n=20) | 147 (31.4) | 31.7 (5.3) | 98.2 (28.7) | 45.4 (5.9) | 18.0 (4.1) | 18.8 (3.4) |
| TD (n=20) | 204 (56.9) | 43.0 (7.6) | 77.2 (31.2) | 34.4 (7.1) | 88.0 (32.4) | 87.8 (19.8) |
| Data are presented as mean and standard deviation. | | | | | | |

**eTable 10. Summary of adverse events (safety population).**

| **Adverse Events** | **Run-in (n=51)** | | **Traditional diet (n=50)** | | **Wash-out, after Trad. (n=25)** | | **Moderate carbohydrate diet (n=51)** | | **Wash-out, after Mod. Carb. (n=27)** | |
| --- | --- | --- | --- | --- | --- | --- | --- | --- | --- | --- |
|  | **Diagnosis** | **Subjects with Diagnosis n (%)** | **Diagnosis** | **Subjects with Diagnosis n (%)** | **Diagnosis** | **Subjects with Diagnosis n (%)** | **Diagnosis** | **Subjects with Diagnosis n (%)** | **Diagnosis** | **Subjects with Diagnosis n (%)** |
| **Any Diagnoses** | 16 | 15 (29.4%) | 27 | 19 (38.0%) | 5 | 2 (8.0%) | 30 | 24 (47.1%) | 11 | 10 (37.0%) |
| **Severe Adverse Events** | 0 | 0 (0.0%) | 0 | 0 (0.0%) | 0 | 0 (0.0%) | 0 | 0 (0.0%) | 0 | 0 (0.0%) |
| **Related (Possible Relationship) Adverse Events** | 0 | 0 (0.0%) | 0 | 0 (0.0%) | 1 | 1 (4.0%) | 2 | 2 (3.9%) | 0 | 0 (0.0%) |
| **Serious Adverse Events** | 0 | 0 (0.0%) | 0 | 0 (0.0%) | 0 | 0 (0.0%) | 0 | 0 (0.0%) | 0 | 0 (0.0%) |

**eTable 11. Adverse events (safety population).**

| **Chapter  Second level** | **Run-in (n=51)** | | **Traditional diet (n=50)** | | **Wash-out, after Trad. (n=25)** | | **Moderate carbohydrate diet (n=51)** | | **Wash-out, after Mod. Carb. (n=27)** | |
| --- | --- | --- | --- | --- | --- | --- | --- | --- | --- | --- |
|  | **Diagnosis** | **Subjects with Diagnosis n (%)** | **Diagnosis** | **Subjects with Diagnosis n (%)** | **Diagnosis** | **Subjects with Diagnosis n (%)** | **Diagnosis** | **Subjects with Diagnosis n (%)** | **Diagnosis** | **Subjects with Diagnosis n (%)** |
|  |  |  |  |  |  |  |  |  |  |  |
| **Any Diagnoses** | **16** | **15 (29.4%)** | **27** | **19 (38.0%)** | **5** | **2 (8.0%)** | **30** | **24 (47.1%)** | **11** | **10 (37.0%)** |
|  |  |  |  |  |  |  |  |  |  |  |
| 01 Certain infectious and parasitic diseases (A00-B99) | **1** | **1 (2.0%)** | **3** | **3 (6.0%)** |  |  |  |  |  |  |
| A08 Viral and other specified intestinal infections |  |  | 2 | 2 (4.0%) |  |  |  |  |  |  |
| B02 Zoster [herpes zoster] |  |  | 1 | 1 (2.0%) |  |  |  |  |  |  |
| B34 Viral infection of unspecified site | 1 | 1 (2.0%) |  |  |  |  |  |  |  |  |
| 04 Endocrine, nutritional and metabolic diseases (E00-E90) |  |  | **2** | **2 (4.0%)** | **1** | **1 (4.0%)** | **3** | **3 (5.9%)** |  |  |
| E10 Type 1 diabetes mellitus |  |  | 2 | 2 (4.0%) | 1 | 1 (4.0%) | 3 | 3 (5.9%) |  |  |
| 05 Mental and behavioral disorders (F00-F99) |  |  |  |  |  |  | **2** | **2 (3.9%)** |  |  |
| F32 Depressive episode |  |  |  |  |  |  | 1 | 1 (2.0%) |  |  |
| F51 Nonorganic sleep disorders |  |  |  |  |  |  | 1 | 1 (2.0%) |  |  |
| 09 Diseases of the circulatory system (I00-I99) |  |  |  |  |  |  | **1** | **1 (2.0%)** |  |  |
| I48 Atrial fibrillation and flutter |  |  |  |  |  |  | 1 | 1 (2.0%) |  |  |
| 10 Diseases of the respiratory system (J00-J99) | **10** | **10 (19.6%)** | **11** | **9 (18.0%)** | **2** | **1 (4.0%)** | **8** | **7 (13.7%)** | **5** | **5 (18.5%)** |
| J00 Acute nasopharyngitis [common cold] | 9 | 9 (17.6%) | 9 | 8 (16.0%) | 2 | 1 (4.0%) | 8 | 7 (13.7%) | 4 | 4 (14.8%) |
| J11 Influenza, virus not identified | 1 | 1 (2.0%) |  |  |  |  |  |  |  |  |
| J30 Vasomotor and allergic rhinitis |  |  | 2 | 2 (4.0%) |  |  |  |  | 1 | 1 (3.7%) |
| 11 Diseases of the digestive system (K00-K93) |  |  | **2** | **2 (4.0%)** |  |  |  |  |  |  |
| K08 Other disorders of teeth and supporting structures |  |  | 1 | 1 (2.0%) |  |  |  |  |  |  |
| K80 Cholelithiasis |  |  | 1 | 1 (2.0%) |  |  |  |  |  |  |
| 12 Diseases of the skin and subcutaneous tissue (L00-L99) | **1** | **1 (2.0%)** | **2** | **2 (4.0%)** | **1** | **1 (4.0%)** | **2** | **2 (3.9%)** | **1** | **1 (3.7%)** |
| L08 Other local infections of skin and subcutaneous tissue |  |  |  |  | 1 | 1 (4.0%) |  |  |  |  |
| L23 Allergic contact dermatitis | 1 | 1 (2.0%) | 1 | 1 (2.0%) |  |  | 2 | 2 (3.9%) | 1 | 1 (3.7%) |
| L60 Nail disorders |  |  | 1 | 1 (2.0%) |  |  |  |  |  |  |
| 13 Diseases of the musculoskeletal system and connective tissue (M00-M99) | **2** | **2 (3.9%)** | **1** | **1 (2.0%)** |  |  | **2** | **2 (3.9%)** | **2** | **2 (7.4%)** |
| M54 Dorsalgia |  |  | 1 | 1 (2.0%) |  |  | 2 | 2 (3.9%) |  |  |
| M60 Myositis | 2 | 2 (3.9%) |  |  |  |  |  |  |  |  |
| M72 Fibroblastic disorders |  |  |  |  |  |  |  |  | 1 | 1 (3.7%) |
| M79 Other soft tissue disorders, not elsewhere classified |  |  |  |  |  |  |  |  | 1 | 1 (3.7%) |
| 14 Diseases of the genitourinary system (N00-N99) | **1** | **1 (2.0%)** |  |  |  |  |  |  |  |  |
| N76 Other inflammation of vagina and vulva | 1 | 1 (2.0%) |  |  |  |  |  |  |  |  |
| 18 Symptoms, signs and abnormal clinical and laboratory findings, not elsewhere classified (R00-R99) |  |  | **4** | **4 (8.0%)** |  |  | **6** | **5 (9.8%)** |  |  |
| R05 Cough |  |  |  |  |  |  | 1 | 1 (2.0%) |  |  |
| R07 Pain in throat and chest |  |  |  |  |  |  | 1 | 1 (2.0%) |  |  |
| R51 Headache |  |  | 1 | 1 (2.0%) |  |  |  |  |  |  |
| R53 Malaise and fatigue |  |  | 3 | 3 (6.0%) |  |  | 4 | 4 (7.8%) |  |  |
| 19 Injury, poisoning and certain other consequences of external causes (S00-T98) | **1** | **1 (2.0%)** | **1** | **1 (2.0%)** | **1** | **1 (4.0%)** | **5** | **5 (9.8%)** | **2** | **2 (7.4%)** |
| S00 Superficial injury of head |  |  |  |  |  |  | 1 | 1 (2.0%) |  |  |
| S05 Injury of eye and orbit |  |  | 1 | 1 (2.0%) | 1 | 1 (4.0%) |  |  |  |  |
| S39 Other and unspecified injuries of abdomen, lower back and pelvis | 1 | 1 (2.0%) |  |  |  |  |  |  |  |  |
| S89 Other and unspecified injuries of lower leg |  |  |  |  |  |  | 1 | 1 (2.0%) | 1 | 1 (3.7%) |
| S97 Crushing injury of ankle and foot |  |  |  |  |  |  | 1 | 1 (2.0%) |  |  |
| S99 Other and unspecified injuries of ankle and foot |  |  |  |  |  |  | 1 | 1 (2.0%) | 1 | 1 (3.7%) |
| T07 Unspecified multiple injuries |  |  |  |  |  |  | 1 | 1 (2.0%) |  |  |
| 21 Factors influencing health status and contact with health services (Z00-Z99) |  |  |  |  |  |  |  |  | **1** | **1 (3.7%)** |
| Z25 Need for immunization against other single viral diseases |  |  |  |  |  |  |  |  | 1 | 1 (3.7%) |
| 22 Codes for special purposes (U00-U99) |  |  | **1** | **1 (2.0%)** |  |  | **1** | **1 (2.0%)** |  |  |
| U12 COVID-19 vaccines causing adverse effects in therapeutic use |  |  | 1 | 1 (2.0%) |  |  | 1 | 1 (2.0%) |  |  |

**eTable 12. Medical and surgical history (Full Analysis Set).**

| **Chapter  Second level** | **Full Analysis Set (n=50)** |
| --- | --- |
| **Any Diagnoses** | **26 (52.0%)** |
|  |  |
| **01 Certain infectious or parasitic diseases (A00-B99)** | **1 (2.0%)** |
| B17 Other acute viral hepatitis | 1 (2.0%) |
| **02 Neoplasms (C00-D48)** | **1 (2.0%)** |
| C50 Malignant neoplasm of breast | 1 (2.0%) |
| D44 Neoplasm of uncertain or unknown behaviour of endocrine glands | 1 (2.0%) |
| **03 Diseases of the blood and blood-forming organs and certain disorders involving the immune mechanism (D50-D89)** | **2 (4.0%)** |
| D50 Iron deficiency anemia | 2 (4.0%) |
| 04 Endocrine, nutritional, and metabolic diseases (E00-E90) | **14 (28.0%)** |
| E03 Other hypothyroidism | 7 (14.0%) |
| E10 Type 1 diabetes mellitus | 3 (6.0%) |
| E78 Disorders of lipoprotein metabolism and other lipidaemias | 11 (22.0%) |
| 05 Mental and behavioural disorders (F00-F99) | **5 (10.0%)** |
| F31 Bipolar affective disorder | 1 (2.0%) |
| F32 Depressive episode | 2 (4.0%) |
| F90 Hyperkinetic disorders | 2 (4.0%) |
| **06 Diseases of the nervous system** **(G00-G99)** | **2 (4.0%)** |
| G40 Epilepsy | 1 (2.0%) |
| G43 Migraine | 1 (2.0%) |
| G56 Mononeuropathies of upper limb | 1 (2.0%) |
| 09 Diseases of the circulatory system (I00-I99) | **13 (26.0%)** |
| I10 Essential (primary) hypertension | 12 (24.0%) |
| I42 Cardiomyopathy | 1 (2.0%) |
| I49 Other cardiac arrhythmias | 1 (2.0%) |
| I64 Stroke, not specified as hemorrhage or infarction | 1 (2.0%) |
| I95 Hypotension | 1 (2.0%) |
| 10 Diseases of the respiratory system (J00-J99) | **4 (8.0%)** |
| J00 Acute nasopharyngitis [common cold] | 1 (2.0%) |
| J45 Asthma | 3 (6.0%) |
| 11 Diseases of the digestive system (K00-K93) | **2 (4.0%)** |
| K40 Inguinal hernia | 1 (2.0%) |
| K90 Intestinal malabsorption | 1 (2.0%) |
| 12 Diseases of the skin and subcutaneous tissue (L00-L99) | **3 (6.0%)** |
| L63 Alopecia areata | 1 (2.0%) |
| L80 Vitiligo | 1 (2.0%) |
| L85 Other epidermal thickening | 1 (2.0%) |
| L92 Granulomatous disorders of skin and subcutaneous tissue | 1 (2.0%) |
| **13 Diseases of the musculoskeletal system and connective tissue (M00-M99)** | **3 (6.0%)** |
| M79 Other soft tissue disorders, not elsewhere classified | 3 (6.0%) |
| 14 Diseases of the genitourinary system (N00-N99) | **2 (4.0%)** |
| N40 Hyperplasia of prostate | 1 (2.0%) |
| N80 Endometriosis of uterus | 1 (2.0%) |
| 19 Injury, poisoning and certain other consequences of external causes (S00-T98) | **1 (2.0%)** |
| S88 Traumatic amputation of lower leg | 1 (2.0%) |
| T02 Fractures involving multiple body regions | 1 (2.0%) |

**eTable 13. Prior Medications (Full Analysis Set).**

| **1st level  4th level** | **Full Analysis Set (n=50)** |
| --- | --- |
| **Any Medication** | **39 (78.0%)** |
|  |  |
| **A Alimentary tract and metabolism** | **7 (14.0%)** |
| A02BC Proton pump inhibitors | 1 (2.0%) |
| A10BA Biguanides | 3 (6.0%) |
| A10BJ Glucagon like peptid-1 -receptor (GLP-1) analogues | 1 (2.0%) |
| A11CC Vitamin D and analogues | 1 (2.0%) |
| A12AA Calcium | 1 (2.0%) |
| A12BA Potassium | 1 (2.0%) |
| **B Blood and blood forming organs** | **7 (14.0%)** |
| B01AC Platelet aggregating inhibitors excl. heparin | 4 (8.0%) |
| B01AX Other antithrombotic agents | 1 (2.0%) |
| B03BA Vitamin B12 (cyanocobalamin and analogues) | 2 (4.0%) |
| **C Cardiovascular system** | **35 (70.0%)** |
| C01A Cardiac glycosides | 1 (2.0%) |
| C03AA Thiazides, plain | 3 (6.0%) |
| C03CA Sulfonamides, plain | 1 (2.0%) |
| C07AB Beta blocking agents, selective | 9 (18.0%) |
| C08CA Dihydropyridine derivatives | 6 (12.0%) |
| C09AA ACE inhibitors, plain | 9 (18.0%) |
| C09CA Angiotensin II receptor blockers (ARBs), plain | 11 (22.0%) |
| C09DA Angiotensin II receptor blockers (ARBs) and diuretics | 3 (6.0%) |
| C10AA HMG CoA reductase inhibitors | 31 (62.0%) |
| C10AB Fibrates | 1 (2.0%) |
| C10AX Other lipid modifying agents | 4 (8.0%) |
| CO7AA Beta blocking agents, non-selective | 1 (2.0%) |
| **G Genito urinary system and sex hormones** | **1 (2.0%)** |
| G04BX Other urologicals | 1 (2.0%) |
| **H Systemic hormonal preparations excl. Sex hormones and insulins** | **14 (28.0%)** |
| H03AA Thyroid preparations | 14 (28.0%) |
| **J Anti-infectives for systemic use** | **1 (2.0%)** |
| J05AB Nucleosides and nucleotides excl. reverse transcriptase inhibitors | 1 (2.0%) |
| **M Musculo-skeletal system** | **4 (8.0%)** |
| M01AE Propionic acid derivatives | 4 (8.0%) |
| **N Nervous system** | **16 (32.0%)** |
| N02AA Natural opium alkaloids | 1 (2.0%) |
| N02AJ Opioids in combination with non-opioid analgesics | 1 (2.0%) |
| N02BE Anilides | 3 (6.0%) |
| N02BF Gabapentinoids | 1 (2.0%) |
| N03AX Other antiepileptics | 1 (2.0%) |
| N04BC Dopaminergics | 1 (2.0%) |
| N05AA Phenothiazines with aliphatic side-chain | 1 (2.0%) |
| N05AH Diazepines, oxazepines, thiazepines and oxepines | 1 (2.0%) |
| N05AN Lithium | 1 (2.0%) |
| N05CM Other hypnotics and sedatives | 1 (2.0%) |
| N06AA Non-selective monoamine reuptake inhibitors | 1 (2.0%) |
| N06AB Selective serotonin reuptake inhibitors | 6 (12.0%) |
| N06AX Other antidepressants | 5 (10.0%) |
| N06BA Centrally acting sympathomimetics | 1 (2.0%) |
| **R Respiratory system** | **5 (10.0%)** |
| R03AA Alpha- and beta-adrenoreceptor agonists | 1 (2.0%) |
| R03AC Selective beta-2-adrenoreceptor agonists | 2 (4.0%) |
| R03AK Adregenics in combination with corticosteroids or other drugs, excl. anticholinergics | 1 (2.0%) |
| R03BA Glucocorticoids | 1 (2.0%) |
| R03CC Selective beta-2-adenoreceptor agonists | 2 (4.0%) |
| R06AX Other antihistamines for systemic use | 1 (2.0%) |
| **S Sensory organs** | **1 (2.0%)** |
| S01EE Prostaglandin analogues | 1 (2.0%) |

**eTable 14. Concomitant Medications (Full Analysis Set).**

| **1st level  4th level** | **Full Analysis Set (n=50)** |
| --- | --- |
| **Any Medication** | **43 (86.0%)** |
|  |  |
| **A Alimentary tract and metabolism** | **7 (14.0%)** |
| SA02BC Proton pump inhibitors | 1 (2.0%) |
| A10BA Biguanides | 3 (6.0%) |
| A10BJ Glucagon like peptid-1 -receptor (GLP-1) analogues | 1 (2.0%) |
| A11CC Vitamin D and analogues | 1 (2.0%) |
| A12AA Calcium | 1 (2.0%) |
| A12BA Potassium | 1 (2.0%) |
| **B Blood and blood forming organs** | **8 (16.0%)** |
| B01AC Platelet aggregating inhibitors excl. heparin | 4 (8.0%) |
| B01AX Other antithrombotic agents | 2 (4.0%) |
| B03BA Vitamin B12 (cyanocobalamin and analogues) | 2 (4.0%) |
| **C Cardiovascular system** | **36 (72.0%)** |
| C01 Cardiac glycosides | 1 (2.0%) |
| C02AC Imidazoline receptor agonists | 1 (2.0%) |
| C03AA Thiazides, plain | 3 (6.0%) |
| C03CA Sulfonamides, plain | 1 (2.0%) |
| C07AB Beta blocking agents, selective | 9 (18.0%) |
| C08CA Dihydropyridine derivatives | 7 (14.0%) |
| C09AA ACE inhibitors, plain | 9 (18.0%) |
| C09CA Angiotensin II receptor blockers (ARBs), plain | 12 (24.0%) |
| C09DA Angiotensin II receptor blockers (ARBs) and diuretics | 3 (6.0%) |
| C10AA HMG CoA reductas inhibitors | 31 (62.0%) |
| C10AB Fibrates | 1 (2.0%) |
| C10AX Other lipid modifying agents | 4 (8.0%) |
| CO7AA Beta blocking agents, non-selective | 1 (2.0%) |
| **G Genito urinary system and sex hormones** | **3 (6.0%)** |
| G01AC Quinoline derivatives | 1 (2.0%) |
| G04BX Other urologicals | 2 (4.0%) |
| **H Systemic hormonal preparations excl. Sex hormones and insulins** | **14 (28.0%)** |
| H02AB Glucocorticoids | 1 (2.0%) |
| H03AA Thyroid preparations | 14 (28.0%) |
| **J Anti-infectives for systemic use** | **1 (2.0%)** |
| J05AB Nucleosides and nucleotides excl. reverse transcriptase inhibitors | 1 (2.0%) |
| **M Musculo-skeletal system** | **7 (14.0%)** |
| M01AB Acetic acid derivatives and related substances | 1 (2.0%) |
| M01AE Propionic acid derivatives | 5 (10.0%) |
| M03BB Oxazole, thiazine, and triazine derivatives | 1 (2.0%) |
| **N Nervous system** | **19 (38.0%)** |
| N02AA Natural opium alkaloids | 1 (2.0%) |
| N02AJ Opioids in combination with non-opioid analgesics | 1 (2.0%) |
| N02BE Anilides | 6 (12.0%) |
| N02BF Gabapentinoids | 1 (2.0%) |
| N03AX Other antiepileptics | 1 (2.0%) |
| N04BC Dopaminergics | 1 (2.0%) |
| N05AA Phenothiazines with aliphatic side-chain | 1 (2.0%) |
| N05AH Diazepines, oxazepines, thiazepines and oxepines | 1 (2.0%) |
| N05AN Lithium | 1 (2.0%) |
| N05CM Other hypnotics and sedatives | 1 (2.0%) |
| N06AA Non-selective monoamine reuptake inhibitors | 1 (2.0%) |
| N06AB Selective serotonin reuptake inhibitors | 6 (12.0%) |
| N06AX Other antidepressants | 5 (10.0%) |
| N06BA Centrally acting sympathomimetics | 1 (2.0%) |
| N07BB Drugs used in alcohol dependence | 1 (2.0%) |
| **R Respiratory system** | **10 (20.0%)** |
| R03AA Alpha- and beta-adrenoreceptor agonists | 2 (4.0%) |
| R03AC Selective beta-2-adrenoreceptor agonists | 2 (4.0%) |
| R03AK Adregenics in combination with corticosteroids or other drugs, excl. anticholinergics | 1 (2.0%) |
| R03BA Glucocorticoids | 1 (2.0%) |
| R03CC Selective beta-2-adenoreceptor agonists | 2 (4.0%) |
| R06AX Other antihistamines for systemic use | 6 (12.0%) |
| **S Sensory organs** | **2 (4.0%)** |
| S01AA Antibiotics | 1 (2.0%) |
| S01EE Prostaglandin analogues | 1 (2.0%) |

**eTable 15.** Post-hoc analysis of mean glucose measured by the area under the curve (AUC) of glucose levels measured by CGM during moderate carbohydrate diet and traditional diet on the full analysis set (FAS) and per-protocol populations (PP1–PP4).

| **Variable / Study population** | **Moderate carbohydrate diet** | **Traditional diet** | **Mean difference (95% CI)** |
| --- | --- | --- | --- |
| **CGM AUC (mmol/L)** |  |  |  |
| **FAS (n=50)** | 8.7 (1.7) | 9.3 (2.1) | -0.7 (-1.0 to -0.3) p<0.001 |
| **PP1 (n=43)** | 8.6 (1.7) | 9.2 (1.9) | -0.6 (-1.0 to -0.2) p=0.002 |
| **PP2 (n=27)** | 8.2 (1.5) | 8.9 (1.8) | -0.8 (-1.3 to -0.2) p=0.015 |
| **PP3 (n=20)** | 8.1 (1.5) | 8.8 (1.9) | -0.7 (-1.5 to 0.1) p=0.091 |
| **PP4 (n=33)** | 8.1 (1.5) | 8.7 (1.7) | -0.6 (-1.1 to -0.2) p=0.008 |
| Data are presented as mean and standard deviation.  Statistical analyses were performed using linear mixed effects models with treatment (diet) and period as fixed effects and subject as random effect.  **Abbreviations:** CI, confidence interval; SD, standard deviation. | | | |
